# Design of template-stabilized active and earth-abundant oxygen evolution catalysts in acid†
†Electronic supplementary information (ESI) available: CVs for unary metal oxides deposition, electrochemical stability at higher current densities for unary metal oxides at pH 2.5, EDS maps for CoMnO_*x*_ and CoPbO_*x*_, STEM images and PXRD of CoMnO_*x*_ and CoFePbO_*x*_, high-resolution XPS of Fe 2p for CoFePbO_*x*_, Pourbaix diagrams (of Mn, Co, Pb, and Fe), and elemental analysis. See DOI: 10.1039/c7sc01239j



**DOI:** 10.1039/c7sc01239j

**Published:** 2017-05-05

**Authors:** Michael Huynh, Tuncay Ozel, Chong Liu, Eric C. Lau, Daniel G. Nocera

**Affiliations:** a Department of Chemistry and Chemical Biology , Harvard University , Cambridge , Massachusetts 02138 , USA . Email: dnocera@fas.harvard.edu

## Abstract

Oxygen evolution reaction (OER) catalysts that are earth-abundant and are active and stable in acid are unknown. Active catalysts derived from Co and Ni oxides dissolve at low pH, whereas acid stable systems such as Mn oxides (MnO_*x*_) display poor OER activity. We now demonstrate a rational approach for the design of earth-abundant catalysts that are stable and active in acid by treating activity and stability as decoupled elements of mixed metal oxides. Manganese serves as a stabilizing structural element for catalytically active Co centers in CoMnO_*x*_ films. In acidic solutions (pH 2.5), CoMnO_*x*_ exhibits the OER activity of electrodeposited Co oxide (CoO_*x*_) with a Tafel slope of 70–80 mV per decade while also retaining the long-term acid stability of MnO_*x*_ films for OER at 0.1 mA cm^–2^. Driving OER at greater current densities in this system is not viable because at high anodic potentials, Mn oxides convert to and dissolve as permanganate. However, by exploiting the decoupled design of the catalyst, the stabilizing structural element may be optimized independently of the Co active sites. By screening potential–pH diagrams, we replaced Mn with Pb to prepare CoFePbO_*x*_ films that maintained the high OER activity of CoO_*x*_ at pH 2.5 while exhibiting long-term acid stability at higher current densities (at 1 mA cm^–2^ for over 50 h at pH 2.0). Under these acidic conditions, CoFePbO_*x*_ exhibits OER activity that approaches noble metal oxides, thus establishing the viability of decoupling functionality in mixed metal catalysts for designing active, acid-stable, and earth-abundant OER catalysts.

## Abstract 2

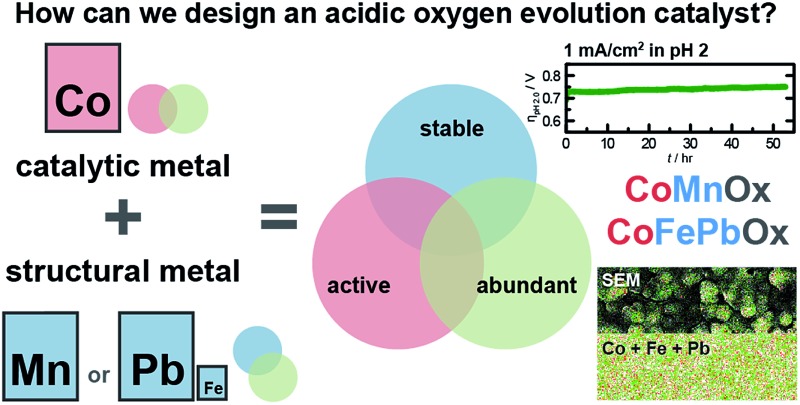
We demonstrate a rational approach for designing earth-abundant catalysts that are stable and active in acid by treating activity and stability as decoupled elements of mixed metal oxides.

## Introduction

Adoption of solar energy is a desired low-risk climate pathway to meet growing global energy demand.^1–6^ Solar energy is the only renewable source scalable to the projected tens of terawatts of global energy consumption while remaining sustainable and cost-effective.^1,2,5,7^ Widespread solar implementation, however, requires inexpensive and high-density energy storage to manage the diurnal nature of the solar energy supply as well as to provide off-grid energy utilization.^5,6^ For large scale storage, batteries are ultimately limited by their energy density, thus providing an impetus for the solar-driven production of chemical fuels; central to this process is the splitting of water to oxygen and hydrogen.^6,8–10^ Of these two reactions, the oxygen evolution reaction (OER) is the more kinetically demanding half-reaction: the transfer of four protons is coupled to the loss of four electrons from two water molecules to produce a single oxygen molecule.^11,12^ We and others have sought to create catalysts that lower these kinetic barriers such that energy can be stored efficiently through water splitting. Inexpensive and active catalysts based on cobalt,^13–16^ nickel,^17–20^ and other earth-abundant metals^21,22^ for OER in neutral and alkaline pH have been developed, but these catalysts corrode in acidic solutions. Recent attempts to realize acid-stable systems from non-critical metals (such as Mn) suffer from low OER activity.^23–28^ Consequently, there are no acidic OER catalysts that are highly active, stable, and earth-abundant with only two out of the three criteria satisfied for known catalysts operating in acid.^29–31^

The acidic pH regime is important for OER because of its applications in electrolysers and photoelectrochemical (PEC) devices as well as in fundamental mechanistic studies and catalyst design.^30–32^ Electrolysers based on acidic proton exchange membranes (PEM) are compact (sharing similar designs as PEM fuel cells), operate at high current and power densities, and achieve low gas crossover. However, their sulfonated Nafion membranes create an acidic local environment of pH ∼0–3 in water which necessitates corrosion-resistant components such as noble metal catalysts comprising Ir and Ru oxides for OER.^33,34^ Similarly, certain PEC photoelectrodes employ photovoltaic materials (such as silicon) that are more stable in acidic pH.^35–38^ Moreover, studying OER in acidic solutions contributes to a mechanistic understanding of how protonation influences OER kinetics when the solution is a poor proton acceptor thus opening avenues for designing and assessing, for example, catalysts with internal proton relays. Finally, the knowledge gained from optimizing catalysts for acid stability may be applied to other reactions at low pH including hydrohalic acid splitting,^39–42^ oxygen reduction (the opposite of OER where good proton donors facilitate the reaction),^43^ and formic/phosphoric acid oxidation (for fuel cell applications).^44^

We now demonstrate a pathway for the design of active, stable, and earth-abundant acidic OER catalysts comprising oxides of mixed metals. The experimental design is based on electrodeposited manganese oxide (MnO_*x*_), which is stable in acid but poorly catalyzes oxygen evolution.^23^ Increased activity for MnO_*x*_ catalysts may be achieved through potential cycling deposition (alternating between anodic and cathodic potentials).^25^ To advance beyond activated MnO_*x*_, we have turned to a design principle to incorporate different co-interacting metals to fulfill specific roles to achieve desired functionality. To this end, OER activity and stability have been decoupled such that each property could be furnished by separate metals and optimized independently. Mixed metal oxide films have been constructed with Co as the catalytic metal and Mn as the structural metal (denoted CoMnO_*x*_). These films exhibit similar catalytic OER activity as electrodeposited cobalt oxide (CoO_*x*_) with Tafel slopes of ∼60 mV per decade in neutral pH and of ∼83 mV per decade in acidic conditions. Whereas CoO_*x*_ fully dissolves within 3 h when operated at 0.1 mA cm^–2^ for OER at pH 2.5, we show that CoMnO_*x*_ remains intact and stable for over 12 h of continuous operation. For the latter, stability at high anodic potential is limited by transformation to permanganate (MnO_4_^–^).^45^ Furthermore, by exploiting the ability to optimize the structural metal independently, Pb oxide was utilized to furnish high anodic stability at low pH. Mixed metal oxides were electrodeposited with Co as the catalytic component and Pb (with Fe dopant) as the structural element. These films exhibit similar Tafel behaviour as CoO_*x*_ in neutral and acidic pH, yet they do not dissolve when operated at current densities of 1 mA cm^–2^ in acid continuously for over 12 h at pH 2.5 and over 50 h at pH 2.0. Under these conditions, the performance of these films begins to approach noble metal oxides, operating at ∼220 mV higher overpotential than Ir oxide. These results demonstrate that catalysts may be designed by mapping their target properties onto individual components of mixed metal films and show that an approach of using a stable metal oxide as a scaffold for active OER metals provides a promising path for the development of active, stable, and earth-abundant OER catalysts.

## Results

### Electrochemical deposition and Tafel analysis

#### Unary metal oxide catalysts

Oxidic thin films containing a single type of metal serve as benchmarks to mixed metal systems composed of Co as the catalytic metal and Mn, Fe, or Pb as the structural elements. Ni and Ir unary oxides are also included for comparison as acid unstable and stable oxides, respectively. For each case, thin metal oxide films (denoted MO_*x*_ where M is the metal) were electrodeposited at constant anodic potential from aqueous solutions containing the metal salt and methylphosphonate (MeP_i_) buffer at pH 8, as is consistent with known procedures for these oxides.^18,24,46,47^ Exceptions to electrodeposition in MeP_i_ include FeO_*x*_ where the kinetics for anodic electrodeposition were slow and thus required bufferless conditions (containing only KNO_3_ as supporting electrolyte) at higher temperatures (75 °C)^48^ and IrO_*x*_ that was electrodeposited from alkaline carbonate buffer.^49^ To determine the anodic deposition potential for each metal oxide film, cyclic voltammograms (CV) were recorded for FTO electrodes in the quiescent precursor solutions (Fig. S1†). By applying potential at the anodic features in the CVs (some of which are described in literature), the following films were deposited: CoO_*x*_ (at a constant anodic potential of 1.05 V), NiO_*x*_ (1.25 V), FeO_*x*_ (1.20 V), MnO_*x*_ (0.54 V), PbO_*x*_ (1.35 V), and IrO_*x*_ (0.85 V). Mass loadings of the films were roughly matched for each sample to account for the different faradaic efficiencies and kinetics of deposition that are unique to each film composition.

**Fig. 1:**
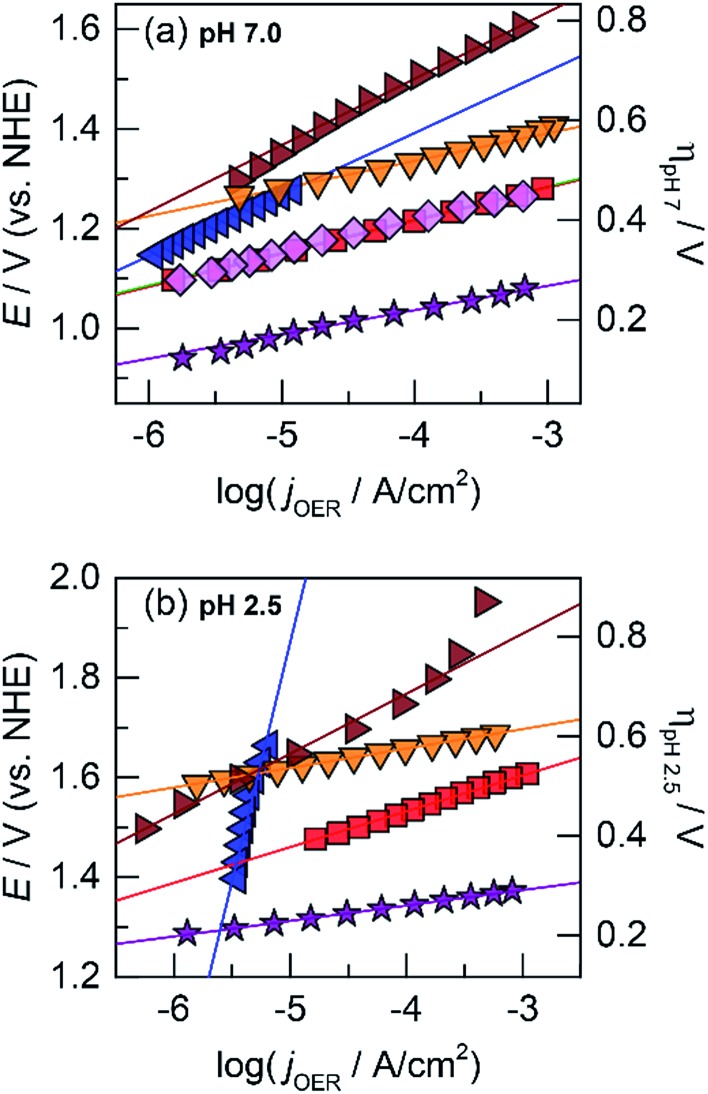
Tafel plots of oxygen evolution for unary metal oxides in 0.10 M P_i_ and 1.0 M KNO_3_ at (a) pH 7.0 and (b) pH 2.5 of CoO_*x*_ (red 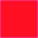, 60 and 82 mV per decade Tafel slope at pH 7.0 and 2.5, respectively), NiO_*x*_ (light magenta 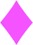, 90 mV per decade at pH 7.0), FeO_*x*_ (orange 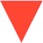, 45 and 51 mV per decade at pH 7.0 and 2.5, respectively), MnO_*x*_ (blue 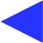, 125 and 650 mV per decade at pH 7.0 and 2.5), PbO_*x*_ (brown 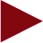, 130 and 121 mV per decade at pH 7.0 and 2.5), and IrO_*x*_ (purple 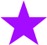, 41 and 32 mV per decade at pH 7.0 and 2.5).

The OER activity of the unary films was evaluated by measuring the steady-state current density as a function of applied potential in a Tafel analysis. Tafel plots for each film were collected in phosphate-buffered (P_i_) solutions at neutral (pH 7.0, Fig. 1a) and acidic (pH 2.5, Fig. 1b) conditions. The slope of these plots served as the primary descriptor for comparing activity because the slope reflects the intrinsic kinetics of the catalyst's active site and is invariant to the amount of deposited catalyst.^50^ At pH 7.0, the Tafel slopes are: 60 mV per decade for CoO_*x*_, 90 mV per decade for NiO_*x*_, 45 mV per decade for FeO_*x*_, 125 mV per decade for MnO_*x*_, 130 mV per decade for PbO_*x*_, and 41 mV per decade for IrO_*x*_. Tafel slopes at pH 2.5 are: 82 mV per decade for CoO_*x*_, 51 mV per decade for FeO_*x*_, 650 mV per decade for MnO_*x*_, 121 mV per decade for PbO_*x*_ (increasing to “infinite” slope at higher current densities), and 32 mV per decade for IrO_*x*_. A reliable Tafel plot could not be constructed for NiO_*x*_ in acid since the film dissolved rapidly in solution. These slopes are consistent with our previous work on Co, Ni, and Mn oxides as well as literature on Fe, Pb, and Ir oxides.^14,18,23,47,51–53^

#### Mixed metal oxide catalysts

**Fig. 2:**
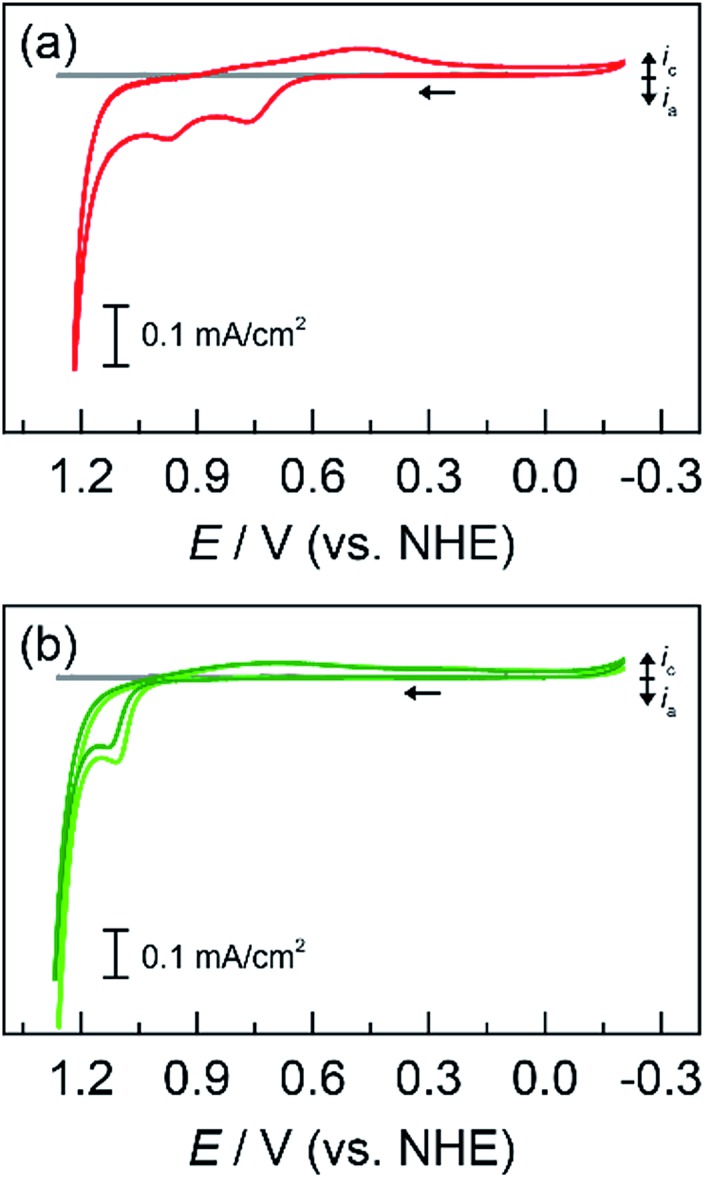
Cyclic voltammograms (CVs) of a 1 cm^2^ FTO electrode at 50 mV s^–1^ in 50 mM MeP_i_ buffer at pH 8.0 with 0.25 mM of each metal: (a) Co^2+^ and Mn^2+^ (red 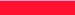); and (b) Co^2+^ and Pb^2+^ (light green 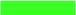) with addition of Fe^2+^ (dark green 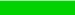). Background CV of metal-free MeP_i_ buffer (grey 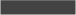) included for comparison.

**Fig. 3:**
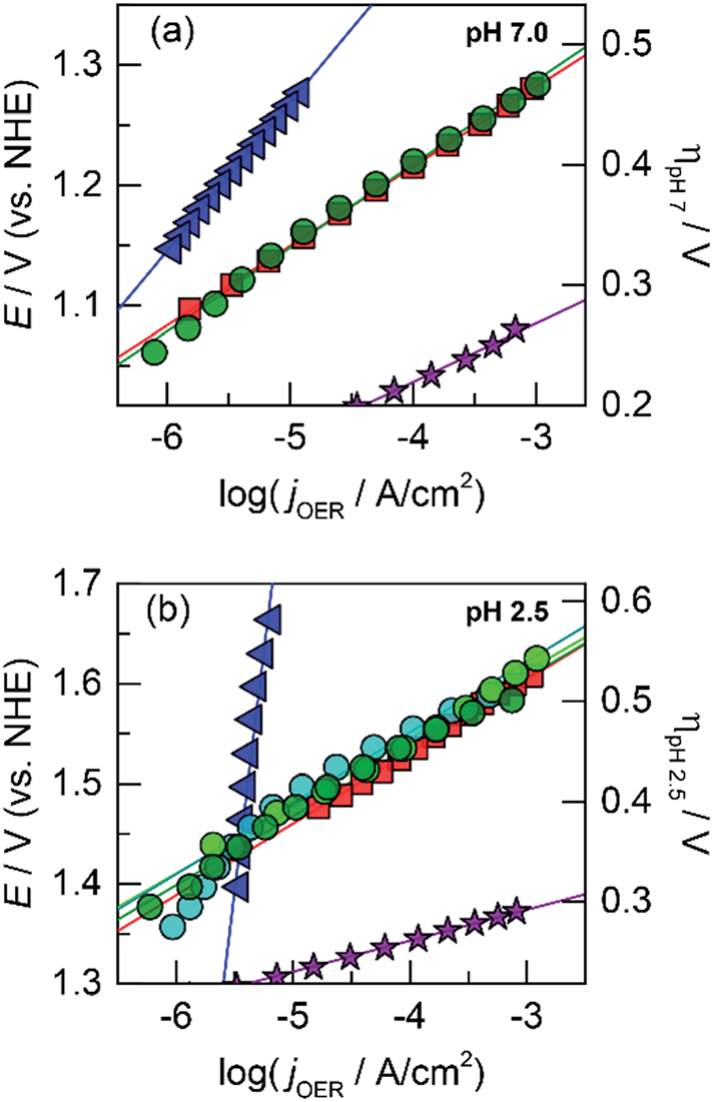
Tafel plots of oxygen evolution for CoMnO_*x*_ in 0.10 M P_i_ and 1.0 M KNO_3_ at (a) pH 7.0 and (b) pH 2.5 of: CoMnO_*x*_ deposited at 0.90 (dark green 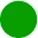, 65 and 81 mV per decade at pH 7.0 and 2.5), 0.65 (light green 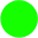, 85 mV per decade at pH 2.5), and 1.15 V (cyan 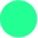, 83 mV per decade at pH 2.5). Unary metal oxides provided for comparison with slopes defined in Fig. 1, CoO_*x*_ (red 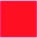), MnO_*x*_ (blue 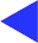), and IrO_*x*_ (purple 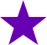).

Having established deposition procedures and kinetic profiles of unary metal oxides, similar steps were taken for mixed metal films. Starting with Co as the catalytic metal and Mn as the structural metal, CVs were collected for solutions containing equal concentration of Co^2+^ with Mn^2+^ (Fig. 2a). The CVs show anodic features similar to that of the individual metals. By comparison to CVs of the native metal ions, the anodic waves at approximately 0.70 and 0.95 V are assigned to MnO_*x*_ and CoO_*x*_ deposition, respectively. Mixed CoMnO_*x*_ films were electrodeposited at three potentials: a light brown film similar to that of MnO_*x*_ was produced near the onset of the MnO_*x*_ deposition wave (0.65 V), a pale brown film formed near the onset of CoO_*x*_ deposition (0.90 V), and a light olive-colored film similar to that of CoO_*x*_ was created past both processes slightly into the catalytic OER wave (1.15 V). Tafel plots in neutral pH (Fig. 3a) show that CoMnO_*x*_ and CoO_*x*_ overlay with similar kinetics behavior of ∼65 mV per decade slope. In acid (pH 2.5), all three cases of CoMnO_*x*_ films were similar to that of CoO_*x*_ with Tafel slopes of ∼83 mV per decade (Fig. 3b). These results suggest that there is no synergistic effect (*i.e.*, improvement of OER activity) between Co and Mn in CoMnO_*x*_ films since the mixed films resemble unary CoO_*x*_, which is catalytically more active than MnO_*x*_ films. The CoO_*x*_-like activity of CoMnO_*x*_ deposited at 0.65 V further suggests that Co is incorporated into the film despite electrodeposition being performed below the qualitative “onset” of CoO_*x*_ formation.

**Fig. 4:**
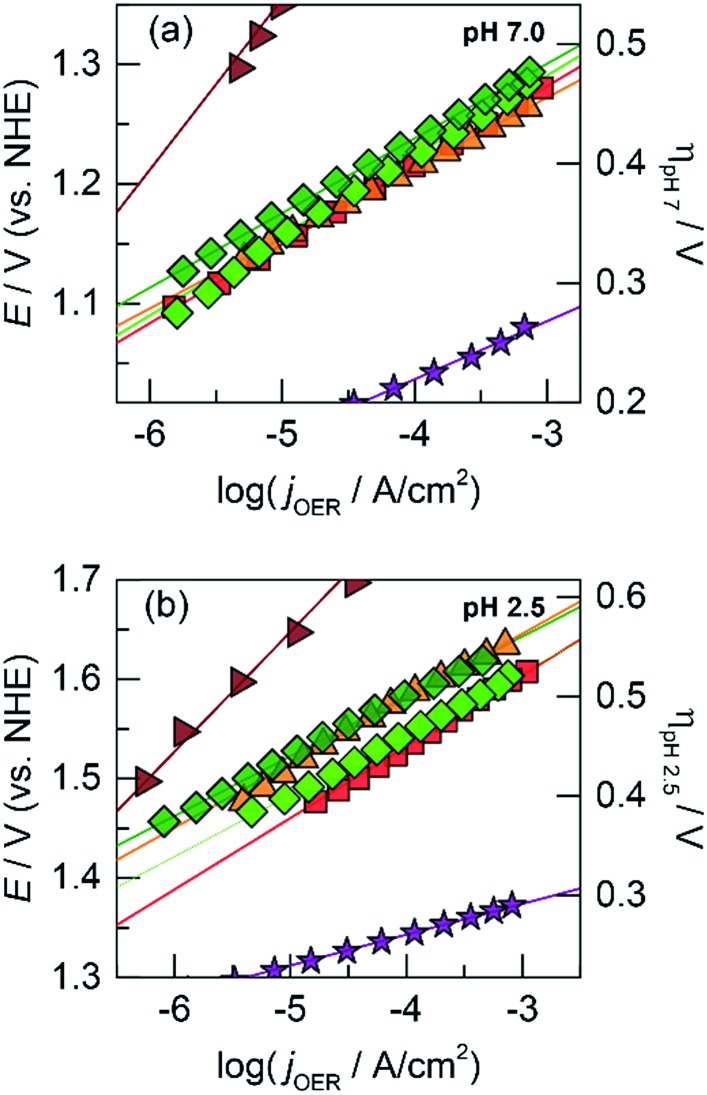
Tafel plots of oxygen evolution in 0.10 M P_i_ and 1.0 M KNO_3_ at (a) pH 7.0 and (b) pH 2.5 for CoPbO_*x*_ (light green 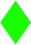, ∼72 mV per decade at both pH 7.0 and 2.5) and CoFePbO_*x*_ (dark green 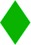, ∼70 mV per decade at both pH 7.0 and 2.5). CoFeO_*x*_ (orange 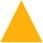, ∼70 mV per decade at both pH 7.0 and 2.5) and unary metal oxides are provided for comparison with slopes defined in Fig. 1, CoO_*x*_ (red 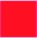), PbO_*x*_ (brown 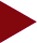), and IrO_*x*_ (purple 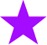).

Lead was substituted for Mn into mixed metal oxides as the structural metal. CVs were collected on solutions with equal concentrations of Co^2+^ with Pb^2+^ (Fig. 2b). For Co^2+^/Pb^2+^ solutions, a CoO_*x*_ deposition pre-feature is observed at 1.15 V; PbO_*x*_ deposition has a broader peak that underlies the pre-feature (>1.1 V according to the CV of PbO_*x*_ in Fig. S1e†). Thus, a fixed constant potential of 1.15 V was employed to electrodeposit brown films of CoPbO_*x*_. Because the deposition of CoO_*x*_ and PbO_*x*_ occurs at similar potentials, it was not possible to control the ratio of mixed metal incorporation in the films by varying the deposition potential. Accordingly, the initial concentrations of the metal ions were adjusted in order to vary metal concentrations within the films. Tafel plots of CoPbO_*x*_ for OER demonstrate kinetics that are similar to that of the unary CoO_*x*_ film. In neutral and acidic pH (Fig. 4a and b), the Tafel slope of CoPbO_*x*_ is ∼72 mV per decade, which is similar to that of CoO_*x*_ and suggests that the mechanism of the active site is the same under a wide range of pH. These results are also consistent with Tafel analysis from CoMnO_*x*_ films and demonstrate that incorporation of structural metals such as Mn or Pb with catalytic metals does not change the OER kinetics of active sites.

Because addition of a structural metal does not interfere with catalytic activity, Pb ions were introduced during the deposition of CoFeO_*x*_ films to assess if the low Tafel slopes of CoFeO_*x*_ in base (∼30 mV per decade)^54^ could translate to neutral and acidic conditions when stabilized by Pb. Using similar deposition solution conditions, CVs were recorded on a solution containing equal concentrations of Co^2+^, Fe^2+^, and Pb^2+^ (Fig. 2b). The lack of a distinct Fe feature in the CVs is consistent with those obtained for Fe^2+^-only solutions (Fig. S1c†), which do not display deposition features due to poor kinetics for anodic FeO_*x*_ formation (in the absence of higher temperatures). Although Fe deposition is not observable in the CV, a small percentage of Fe is trapped and incorporated into the mixed films during growth as supported by elemental analysis (*vide infra*) and studies on similar NiFeO_*x*_ systems.^20,55^ Tafel plots of the Fe-incorporated mixed films (CoFePbO_*x*_ films, Fig. 4a and b) at neutral and acidic pH exhibit similar slopes of ∼70 mV per decade as obtained for Fe-free films. The highly facile kinetics of CoFeO_*x*_ in the alkaline regime with 30 mV per decade slope does not appear to persist in neutral and acidic solutions.

### Acid stability and faradaic efficiency

**Fig. 5:**
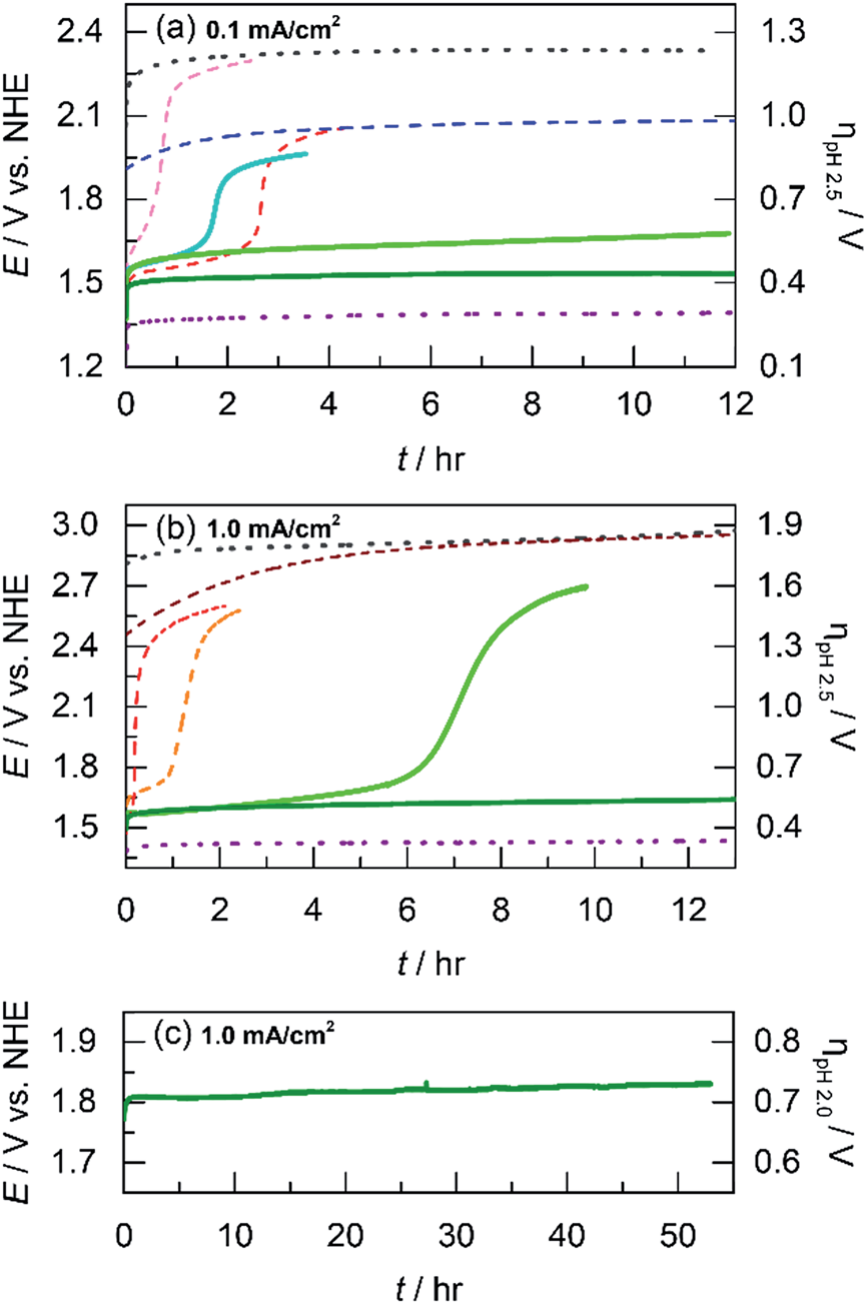
Electrochemical stability for acidic OER measured by sustained chronoamperometry at: (a) 0.1 mA cm^–2^ in pH 2.5 P_i_ for CoMnO_*x*_ deposited at 0.65 (light green 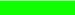), 0.90 (dark green 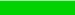), and 1.15 V (cyan 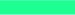) along with CoO_*x*_ (red 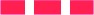), NiO_*x*_ (light magenta 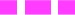), MnO_*x*_ (blue 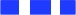), IrO_*x*_ (purple 
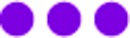
), and FTO (grey 
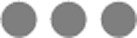
) for comparison; (b) 1.0 mA cm^–2^ in pH 2.5 P_i_ for CoPbO_*x*_ (light green 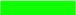) and CoFePbO_*x*_ (dark green 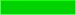) with CoFeO_*x*_ (orange 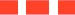), CoO_*x*_ (red 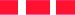), and PbO_*x*_ (brown 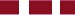) for comparison; and (c) 1.0 mA cm^–2^ in pH 2.0 sulfate for CoFePbO_*x*_ (dark green 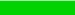). The inflection of potential in the plots indicates film dissolution.

The acidic stability of unary and mixed metal films was evaluated by using long-term chronopotentiometry. Solution conditions were the same as that employed for Tafel analysis (*i.e.*, phosphate buffer with nitrate supporting electrolyte at pH 2.5). Electrodeposited films were immersed in stirred solutions and initially held at a constant current density of 0.1 mA cm^–2^ where after a brief capacitance period, only OER is sustained (as confirmed by direct O_2_ measurement, *vide infra*). OER on blank FTO occurs at a steady potential of 2.3 V (shown for ∼12 h in Fig. 5a). Whereas the substrate oxide is highly acid stable, it is an exceptionally poor OER catalyst. NiO_*x*_ films dissolve quickly at pH 2.5. The applied potential required to maintain 0.1 mA cm^–2^ rises immediately and undergoes inflection at 40 min to correspond to the potential of that for blank FTO, indicating complete film dissolution. CoO_*x*_ films have slightly longer stability than NiO_*x*_ and catalyze OER at ∼1.6 V for almost 3 h before potential inflection, a sign of full dissolution. We note that dissolved Co^2+^ ions can still promote OER^56^ and thus the potential plateaus about 300 mV below that of blank FTO. Moving to the left on the periodic table, we have shown that MnO_*x*_ can perform OER in acid with limited degradation by trading off activity for stability.^23^ Indeed whereas MnO_*x*_ requires a high anodic potential of 2.0 V to maintain 0.1 mA cm^–2^, the current remains stable for over 12 h of testing. As a control, IrO_*x*_ is included to represent a catalyst with both high activity and stability. OER from IrO_*x*_ occurs at 1.4 V sustaining 0.1 mA cm^–2^ over the 12 h testing duration.

To investigate if a structural metal could stabilize unary metal oxides against dissolution, Co (the catalytic element) was co-deposited with Mn (the structural component) at three different potentials (0.65, 0.90, and 1.15 V) to create CoMnO_*x*_ films with different Co : Mn ratios. Stability tests were repeated for these films under the same conditions as for unary catalysts (Fig. 5b). For OER at 0.1 mA cm^–2^, CoMnO_*x*_ electrodeposited at 0.90 V exhibited a low OER potential of 1.5 V (similar to that of CoO_*x*_ before dissolution) while remaining intact over 12 h of operation. Similarly, for CoMnO_*x*_ deposited at 0.65 V, the films remained stable but exhibited a slight increase of 100 mV in its potential to sustain OER. In contrast, films deposited at 1.15 V were not stable and dissolved in ∼2 h, similar to the CoO_*x*_-only film. Overall, mixed metal films containing Co as the catalytic and Mn as the structural element can improve acid stability, extending it to over 12 h, while retaining the OER activity of CoO_*x*_.

While CoMnO_*x*_ is stable for acidic OER at 0.1 mA cm^–2^, higher current densities are desired for practical applications and so the unary and mixed metal films were re-evaluated at 1 mA cm^–2^ under the same solution conditions (Fig. S2†). As a baseline, blank FTO is stable but requires a high potential of ∼2.9 V for OER. CoMnO_*x*_ films (deposited at 0.90 V) initially evolve oxygen at ∼1.7 V but fully dissolve after 30 min. After dissolution, OER is catalyzed by free Co^2+^ ions at ∼2.5 V. For comparison, CoO_*x*_ exhibits similar behavior but fully degrades slightly faster at 20 min. MnO_*x*_ reaches dissolution in 10 min, and the solution color turns slightly pink, which is consistent with the oxidation of the film to permanganate (MnO_4_^–^) as predicted by the Pourbaix diagram of manganese.^45^ The MnO_4_^–^ ions appear to catalyze OER at ∼2.4 V. In contrast, IrO_*x*_ is both stable and highly active, performing OER without degradation at 1.43 V sustaining 1 mA cm^–2^ over 12 h.

The dissolution of Mn at high anodic potentials (caused by operating at higher current densities) prompted its replacement by another structural metal that is not prone to molecular decomposition. Lead was selected as an alternative for manganese after evaluating the potential–pH properties of many corrosion-resistant elements (search process presented in the Discussion). As a control, acidic stability tests at 1 mA cm^–2^ were performed on PbO_*x*_, which demonstrated long-term acidic stability over 12 h at 2.9 V (Fig. 5b). Because Pb was a promising structural metal, mixed metal films incorporating Co and Pb were prepared and evaluated under similar conditions at higher current densities. Indeed, the presence of Pb in CoPbO_*x*_ films extended the operation of “CoO_*x*_” from 20 min to 7 h at ∼1.65 V before eventually dissolving (Fig. 5b). Pb thus slowed the rate of corrosion in CoPbO_*x*_ films significantly.

Incorporation of lead was also investigated in cobalt oxide films doped with Fe. As a control, CoFeO_*x*_ degrades quickly during acidic OER at 1 mA cm^–2^ as indicated by the rising potential around 1.7 V that inflects to indicate dissolution after 1 h (Fig. 5b). The unary FeO_*x*_ is also unstable at higher current densities, degrading after 2 h (Fig. S2†). However, the slight addition of Fe to CoPbO_*x*_ produced CoFePbO_*x*_ films that could sustain OER at ∼1.65 V for over 12 h. Moreover, CoFePbO_*x*_ operated at 1 mA cm^–2^ in sulfate buffer at pH 2.0 (Fig. 5c) remained stable at ∼1.8 V for over 50 h of continuous electrolysis, demonstrating that these films are both active and exhibit superior stability under laboratory test conditions. Overall, Pb appears to fulfill its role as a structural element and confers acidic corrosion resistance when incorporated in mixed metal oxide films.

To confirm that acid-stable mixed metal films are performing OER, the evolved oxygen was directly measured by gas chromatography in a gas-tight electrochemical cell and compared to the charge passed to the electrode during electrolysis. Employing the same solution conditions as that used for stability tests, CoMnO_*x*_ was operated at 0.1 mA cm^–2^ and CoFePbO_*x*_ at 1.0 mA cm^–2^, and the O_2_ concentration was measured every 20 min (Fig. S3†). The faradaic efficiency was calculated by dividing the moles of detected oxygen by the moles of transferred electrons. The average efficiency for CoMnO_*x*_ is 91% and that for CoFePbO_*x*_ is 97%. The slight decrease in efficiency of CoMnO_*x*_ may result from the lower current densities of operation that correspond to a lower O_2_ signal-to-noise ratio and consequently decreased accuracy. Overall, both systems demonstrate faradaic efficiencies near unity for long-term OER in acid.

### Physical characterization

#### Elemental analysis

To determine how deposition protocols affect the composition of mixed metal oxide films, elemental analysis of the films were performed by energy dispersive X-ray spectrometry (EDS) and were confirmed on select samples with inductively coupled plasma mass spectrometry (ICP-MS). EDS was first explored for CoMnO_*x*_ films deposited at 0.65 (near the onset of the MnO_*x*_ deposition but below that of CoO_*x*_ deposition), 0.90 (near the onset of CoO_*x*_ deposition), and 1.15 V (past both processes and slightly into the catalytic OER wave). The EDS spectra were quantified using ZAF (atomic number, absorption, and fluorescence) correction factors to obtain the relative ratios of elements (Table S1†). The composition for films deposited at 0.65 V is 40% Co and 60% Mn, 0.90 V is 50% each of Co and Mn, and 1.15 V is 83% Co and 17% Mn. EDS results for CoMnO_*x*_ deposited at 0.90 V were examined by ICP-MS, a more rigorous technique for elemental analysis, and revealed a composition of 51% Co and 49% Mn, which is consistent with EDS reported values. These experiments demonstrate that the composition of CoMnO_*x*_ films can be controlled by the deposition potential—as potential increases (to the direction of CoO_*x*_ formation), more Co is incorporated in the mixed films.

For mixed films containing Pb, ICP-MS shows that CoPbO_*x*_ comprises 18% Co and 82% Pb, while CoFePbO_*x*_ contains 15% Co, 2% Fe, and 83% Pb (Table S1†). In these films, the structural element (Pb) is the dominant component. Nevertheless, co-depositions include appreciable amounts of catalytic sites and sufficient Fe infiltrates the films during growth, despite the difficulty of directly electrodepositing FeO_*x*_ at those same potentials. Overall, elemental analysis shows that anodic co-deposition is a viable technique for incorporating both catalytic and structural elements in the resulting mixed metal films.

#### Morphology and homogeneity

**Fig. 6:**
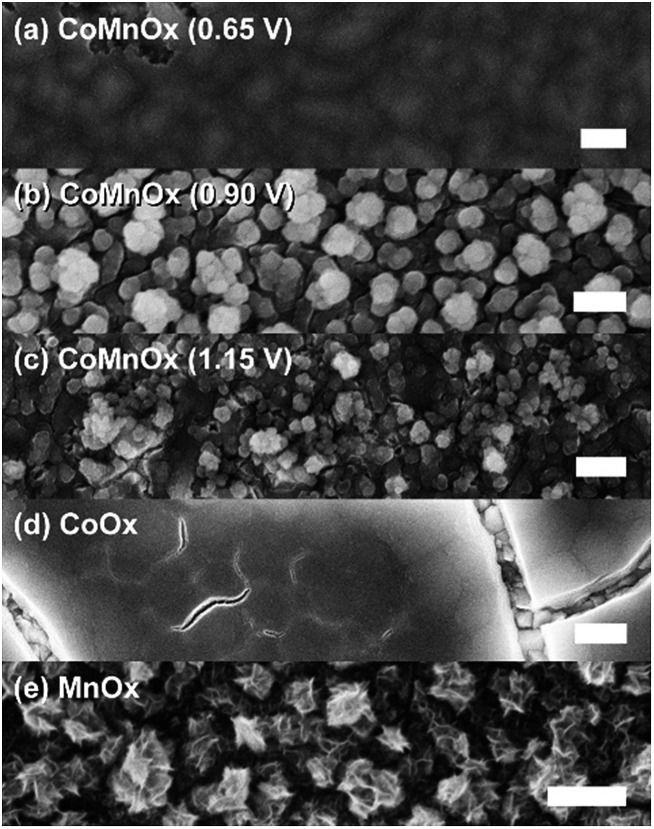
FESEM images of CoMnO_*x*_ electrodeposited at (a) 0.65, (b) 0.90, and (c) 1.15 V with (d) CoO_*x*_ and (e) MnO_*x*_ for comparison. All samples were prepared on FTO substrate, and scale bars are 200 nm.

**Fig. 7:**
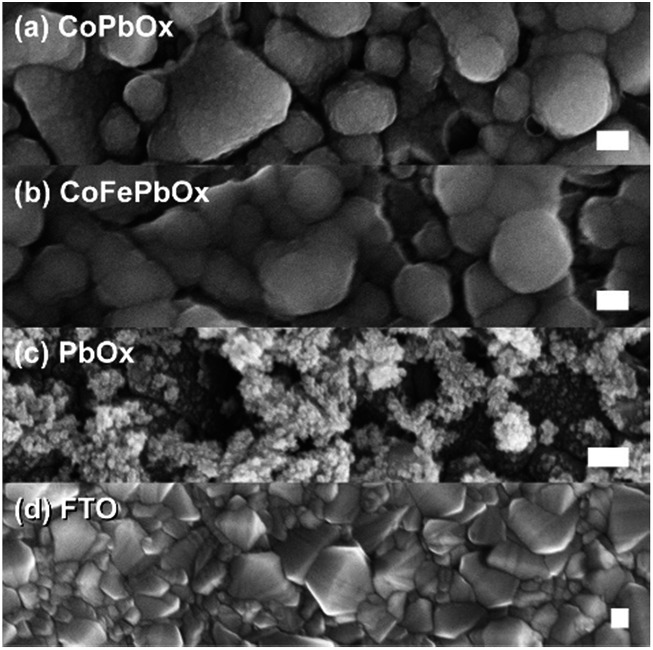
FESEM images of (a) CoPbO_*x*_ and (b) CoFePbO_*x*_ with (c) PbO_*x*_ and (d) FTO for comparison. All samples were electro-deposited on FTO, and scale bars are 100 nm.

**Fig. 8:**
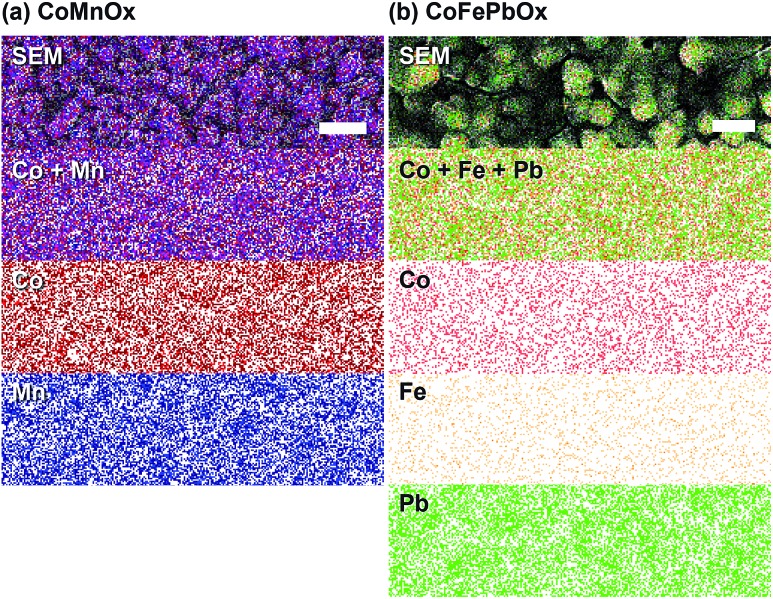
EDS elemental maps recorded through SEM of (a) CoMnO_*x*_ (deposited at 0.90 V) and (b) CoFePbO_*x*_. Individual elemental channels for Co (red), Mn (blue), Fe (orange), and Pb (green) were combined and overlaid on the respective SEM image. All samples were prepared on FTO substrate, and scale bars are 200 nm for CoMnO_*x*_ and 100 nm for CoFePbO_*x*_.

**Fig. 9:**
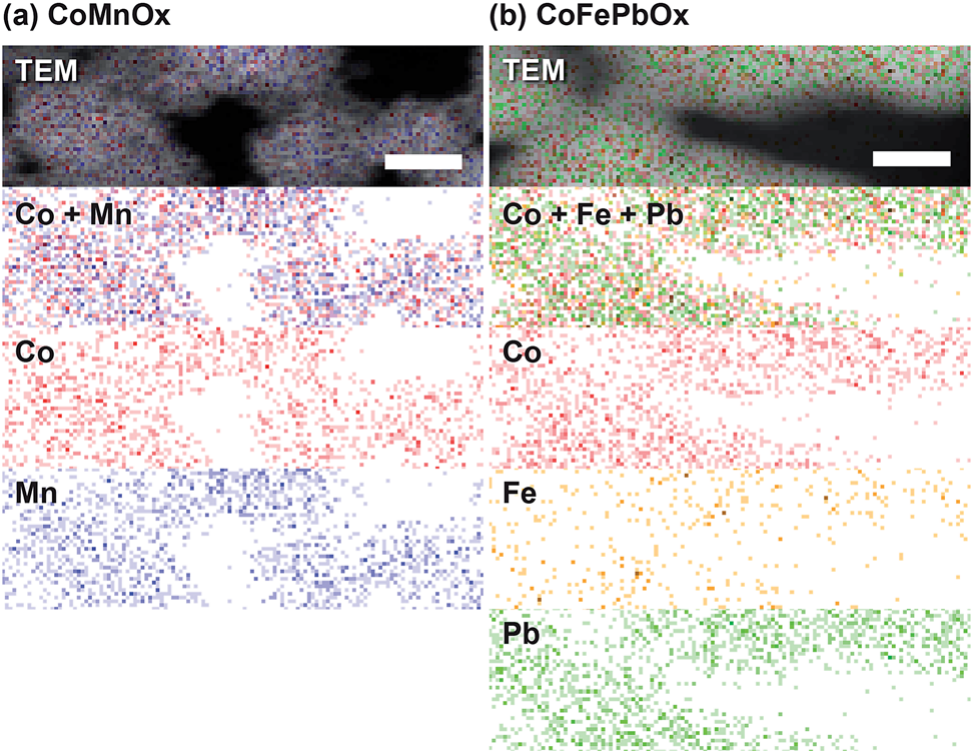
High-resolution EDS elemental maps recorded through STEM of (a) CoMnO_*x*_ (deposited at 0.90 V) and (b) CoFePbO_*x*_. Individual elemental channels for Co (red), Mn (blue), Fe (orange), and Pb (green) were combined and overlaid on the respective image. Scale bars are 15 nm for a resolution of 7.4 Å per px.

Scanning electron microscopy (SEM) was used to investigate homogeneity of mixed metal films (Fig. 6 and 7); images of unary metal oxide catalyst are provided as reference. CoO_*x*_ has a smooth surface with slight cracking from drying, MnO_*x*_ exhibits thin filament-like petals, and PbO_*x*_ has ∼10 nm particles that combine into larger porous structures. For CoMnO_*x*_ films (Fig. 6), deposition at 0.65 V results in a smooth sheet, 0.90 V produces round grains that are aggregates of even smaller 100–200 nm diameter particles, and 1.15 V gives a mixture of sheet and ball features. Similarly, CoPbO_*x*_ shows formation of 100–200 nm particles, and CoFePbO_*x*_ resembles CoPbO_*x*_ in that there are nanoparticles (slightly larger) that merge together to create a continuous coating (Fig. 7). Elemental maps by SEM (EDS) were constructed for each case and show that CoMnO_*x*_ and CoFePbO_*x*_ films are homogeneously mixed on the 20 nm per pixel resolution (Fig. 8 and S4†). Because it is possible for mixed metals to form separate individual metal oxide domains that have smaller than 20 nm diameter, scanning transmission electron microscopy (STEM) with EDS was employed for CoMnO_*x*_ and CoFePbO_*x*_ films on the angstrom-level resolution (Fig. 9). The lack of coherent lattice fringes in the STEM images indicates that the catalyst is largely amorphous (Fig. S5†). Importantly, EDS maps on the 7.4 Å per pixel resolution further demonstrate homogeneous distribution of Co throughout the structural metals (Mn or FePb). Overall, high-resolution electron microscopy coupled with positional elemental mapping suggest that the electrodeposited mixed films incorporate catalytic and structural metals homogeneously on the length scale of these experiments.

#### Powder X-ray diffraction

To check if the mixed metal oxide films form crystalline phases that could lead to the identity of the film composition, Bragg–Brentano X-ray diffraction was performed on powders of CoMnO_*x*_ and CoFePbO_*x*_. These powders were prepared by thin film electrodeposition on large FTO substrates. The films were then mechanically separated from the underlying FTO and ground into a powder. The advantage of this approach is that sufficient catalyst material may be accumulated to obtain better diffraction signal and that the strongly interfering FTO substrate may be eliminated. Both CoMnO_*x*_ and CoFePbO_*x*_ films exhibited no features over a wide range of 2*θ*, with only a strong baseline that is indicative of amorphous material (Fig. S6†). These results are consistent with STEM imaging where catalyst domains lack lattice fringes.

#### Oxidation state

**Fig. 10:**
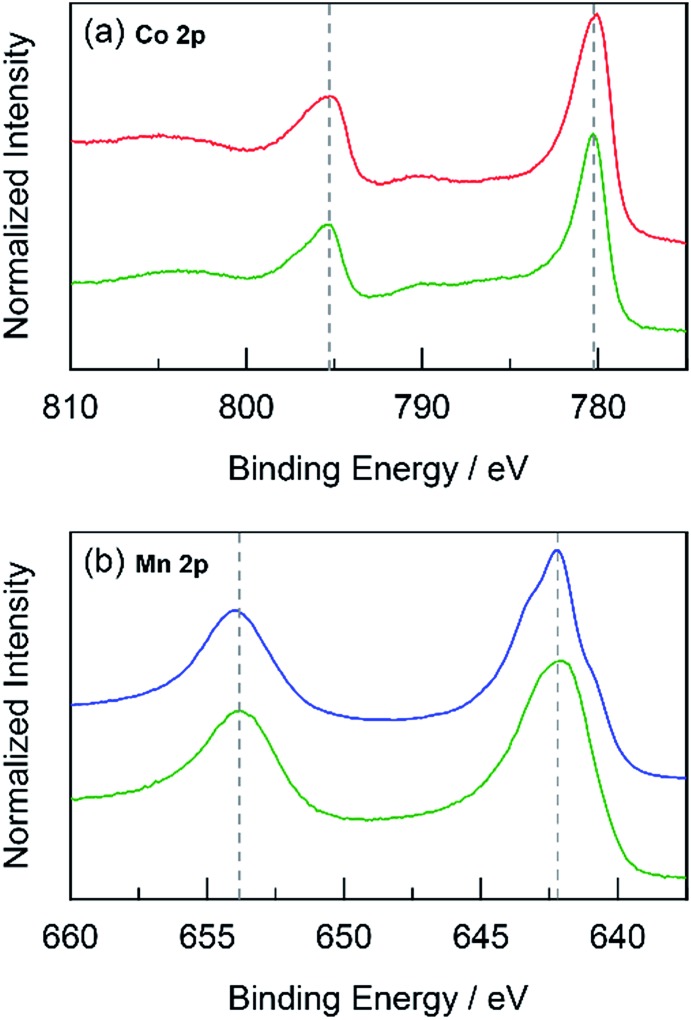
High-resolution XPS spectra in the (a) Co 2p and (b) Mn 2p regions for: CoMnO_*x*_ (deposited at 0.90 V, dark green 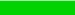) compared to CoO_*x*_ (red 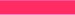), and MnO_*x*_ (blue 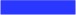). Grey dotted lines are presented as guides.

**Fig. 11:**
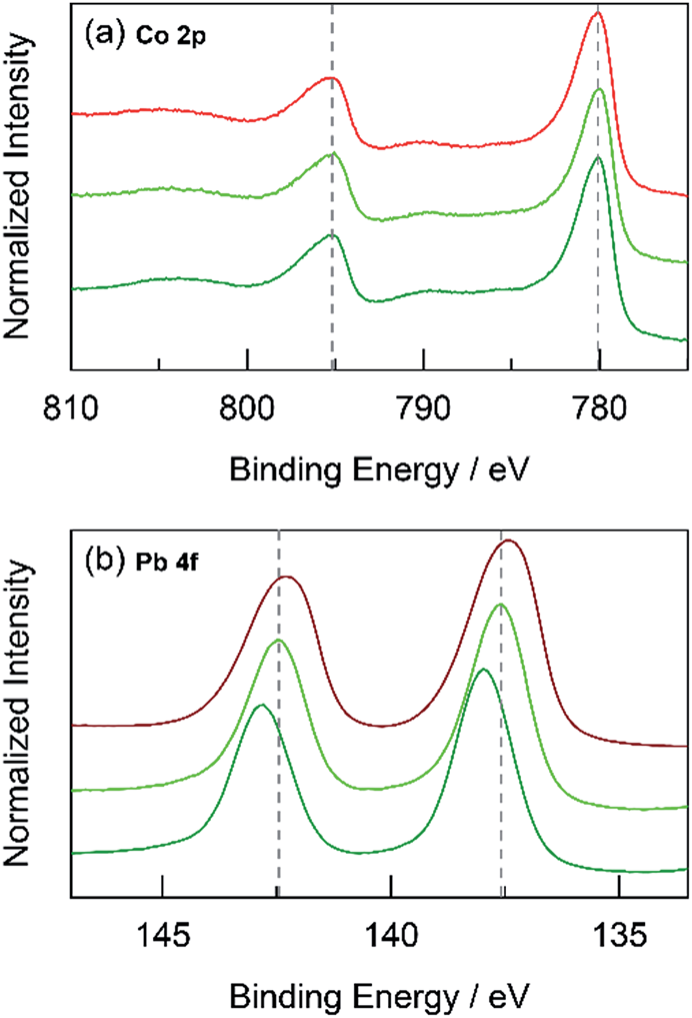
High-resolution XPS spectra in the (a) Co 2p and (b) Pb 4f regions for: CoPbO_*x*_ (light green 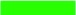) and CoFePbO_*x*_ (dark green 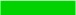) compared to CoO_*x*_ (red 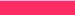), and PbO_*x*_ (brown 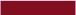). Grey dotted lines are presented as guides.

X-ray photoelectron spectroscopy (XPS) was employed to study any differences in the oxidation state between unary and mixed metal oxide catalysts. Thin films were electrodeposited on FTO and high-resolution scans for each metal in the film were recorded. Because metal oxides are generally poor conductors, any excess surface charge that may accumulate during XPS was neutralized by charge compensation through a low-energy (0–14 eV) electron flood gun. The compensation was effective and preserved the high-precision of XPS since all resulting adventitious C 1s peaks were located within a standard deviation of ∼0.05 eV. The C 1s peaks were finally referenced to a standard value of 284.8 eV,^57^ which shifts all peaks by a small constant offset to account for any remaining uncorrected surface charge. For CoMnO_*x*_ deposited at 0.90 V (composed of 50% each Co and Mn), the Co 2p and Mn 2p regions were compared to that of CoO_*x*_ and MnO_*x*_, respectively (Fig. 10). The Co 2p spectra were Co_3_O_4_-like^58^ and analogous in peak positions and shape between both CoMnO_*x*_ and CoO_*x*_ samples. The Mn 2p spectra for both samples were also similar, but CoMnO_*x*_ has a slightly broader 2p_3/2_ peak than MnO_*x*_, which suggests a decrease of the peak at 642.2 eV with an increase at 643.5 eV. Since these features reflect populations of Mn in different chemical states, growth at the higher peak binding energy supports an average Mn oxidation state closer to +4.0 (or β-MnO_2_), which is inactive for OER.^25^ CoPbO_*x*_ and CoFePbO_*x*_ films also demonstrate no change in the Co 2p positions or shape when compared to CoO_*x*_ (Fig. 11). However, the Pb 4f region exhibits an overall increase of ∼0.6 eV in binding energy progressing from PbO_*x*_ to CoPbO_*x*_ to CoFePbO_*x*_. The presence of Co and additional doping of Fe appears to maintain a high average Pb oxidation state similar to that of PbO_2_.^59^ The trace amount of Fe in CoFePbO_*x*_ films (comprising 2% of the film by ICP-MS) was too low for detection by XPS, especially since the Fe 2p peaks overlap with the broad Sn 3p_3/2_ feature that manifests from the FTO substrate (Fig. S7†). Overall, XPS suggests that there is no change in Co sites when co-deposited with Mn or Pb, which is consistent with results from Tafel analysis showing no difference in catalysis between unary and mixed metal systems.

## Discussion

Heterogeneous OER catalysts based on metal oxides exhibit a propensity for corrosion in low pH solutions. Acids react with basic metal oxides to protonate the metal oxide framework, weakening metal–oxygen bonds and facilitating dissolution.^60–62^ For this reason, the only known viable acidic OER catalysts are based on platinum group metals, in particular, iridium and ruthenium.^31,63,64^ Consequently, performing OER in acid typically relies on using catalysts derived from Ir and Ru oxides with most studies focused on increasing the accessible active sites while decreasing the mass loading.^65–69^ Conversely, development of OER catalysts from earth-abundant metal oxides have targeted the alkaline regime, where stability is generally not a concern, with the use of multiple metals for the sole purpose of catalytic synergy (*e.g.* NiFe oxides) or for increasing the surface area of active catalyst material.^70–74^ OER in neutral solutions has been achieved through the development of self-healing unary catalysts,^75,76^ and in some cases, performance in acid may be achieved at low current density.^23,25^ Our interest to develop further design principles for active, stable, and earth-abundant acidic OER catalysts has led us to consider using mixed-metals as a framework for decoupling activity from stability by employing two different metals: one that acts as a catalytic element and the other that acts as a structural element. In assessing these mixed-metal catalysts, their performance is benchmarked against each other and to unary metal oxide films using techniques employed in our previous mechanistic studies^14,19,25^ as well as those outlined elsewhere.^29,77–79^ Benchmarking typically comprises measurements on elemental composition, surface area, faradaic efficiency, catalytic activity, and stability. Of these criteria, we exclude surface area because a rigorous measurement on porous thin films is unfeasible.^80^ Instead we rely on activity comparisons as defined by Tafel plots, where the slope is a measure of catalyst kinetics that is independent of catalyst loading or surface area.

**Fig. 12:**
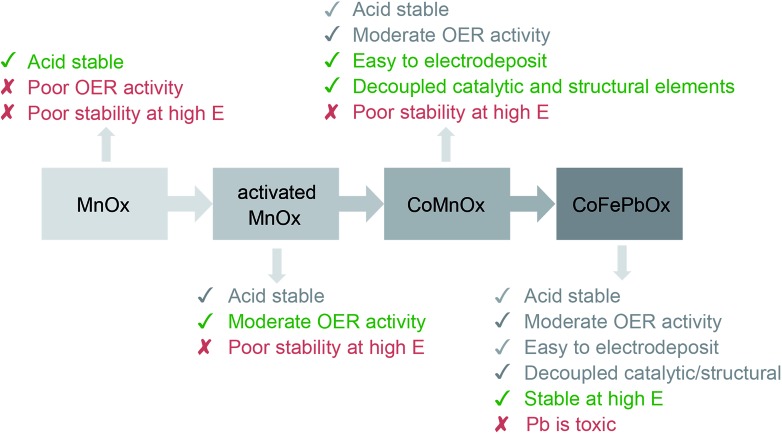
Progression of designing an active, stable, and earth-abundant acidic OER catalyst. The first generation system focused on demonstrating film stability at low pH with MnO_*x*_.^23,24^ The activity of MnO_*x*_ was improved in the second generation by activating MnO_*x*_ for OER.^25^ The catalyst was reformulated as a mixed metal oxide for the third generation, where functionality was separated into catalytic and structural elements comprising Co and Mn, respectively. Finally, the degradation of Mn oxides at high anodic potentials was solved by replacing it with a FePb oxide structural component to create the fourth-generation catalyst.

The iterative design path employed for this study is shown in Fig. 12. Metal oxidic films increase in stability from NiO_*x*_ to CoO_*x*_ and to MnO_*x*_, a trend which is consistent with an increase in metal–oxygen bond strength traversing from late to early first-row transition metals as embodied by the principles of the “oxo wall”.^81^ Indeed, the intrinsic strength of Mn–O bonds in MnO_*x*_ results in resistance to corrosion of the oxide at low pH; this passive stability is augmented by the functional stability of the film derived from self-healing (the catalyst can re-form at the anodic potentials of OER^23,24^). Nonetheless, the greater stability of MnO_*x*_ occurs at the expense of lower activity owing to the formation of MnO_4_^–^ at the higher anodic potentials that accompany higher activity. The activity of conventionally electrodeposited MnO_*x*_ films may be improved^82–84^ by driving a phase change from a birnessite-like (δ-MnO_2_) to hausmannite-like (α-Mn_3_O_4_) oxide that culminates in metastable turbostratic-disordered birnessite.^25^ This phase (denoted activated MnO_*x*_) is significantly more active for OER at pH 2.5 (than unactivated MnO_*x*_) with faster kinetics (*i.e.*, a lower Tafel slope of ∼90 mV per decade and 100 times greater current density at *η* = 600 mV) while remaining stable for over 8 h of continuous operation at 0.1 mA cm^–2^. Spectroscopic and structural studies suggest that these phase changes trap catalytic Mn^III^ sites within a robust Mn^IV^ oxidic matrix, and thus there are two types of Mn sites that allow both activity and stability to co-exist.^25,85,86^ However with structure and function embodied in the same metal, there is no clear path for independent optimization.

### CoMnO_*x*_

Accordingly, we turned to using manganese as the structural element and Co as the catalytic element as we have extensively characterized CoO_*x*_, which is among the most active materials for OER in neutral pH.^87^ An advantage of a mixed metal Mn and Co system is that the oxides can be easily electrodeposited at modest potentials in buffered solutions (MeP_i_ at pH 8) with known deposition kinetics.^24,46^ Furthermore, the ratio of Co to Mn in the films can be controlled by the deposition potential. In the CV of Co^2+^ and Mn^2+^ in MeP_i_ buffer at pH 8 (Fig. 2a), the deposition peak for MnO_*x*_ occurs ∼250 mV to lower anodic potential (*i.e.*, less positive) than that of CoO_*x*_ deposition. This separation in potentials can be exploited to regulate the kinetics of the two reactions. When the electrode potential is near that of MnO_*x*_ deposition, more Mn than Co will be incorporated into the catalyst. As the potential is raised nearer to that of CoO_*x*_ deposition, more Co is assimilated into the final film (Table S1†). SEM and STEM elemental mapping, to resolution of ∼7.4 Å, indicates no segregated domains of CoO_*x*_ and MnO_*x*_ (Fig. 8a and 9a).

Whereas CoMnO_*x*_ has utility in battery and supercapacitor materials^88,89^ and has been studied for seawater^90^ and alkaline^91^ electrolysis, their application towards acidic OER has only recently begun to be explored through screening.^78^ For the studies performed herein, the performance of CoMnO_*x*_ for oxygen evolution in neutral and acidic pH was evaluated using Tafel analysis (*i.e.*, log *j vs. E* plots). In general, better catalysts are positioned lower on the Tafel plots (*i.e.*, requiring less potential to achieve the same current density) and have lower slopes (*i.e.*, produces more current density at the same potential). However, the position of the plot is dictated by the exchange current density (*i.e.*, the *y*-intercept), which is dependent on the number of active sites in the catalyst films. Because there are no known methods to quantify active sites accurately for heterogeneous systems (with the exception of single crystal substrates),^80^ we prefer to rely on the Tafel slope, which reflects the kinetics or mechanism of the reaction and is governed by the type of active site rather than its abundance.^50,92^ Both CoMnO_*x*_ and CoO_*x*_ exhibit the same slope in neutral (∼65 mV per decade) and acidic pH (∼82 mV per decade) suggesting that it is the Co sites in the mixed films that are supporting OER catalysis. Furthermore, given that MnO_*x*_ has worse OER kinetics at neutral (125 mV per decade) and acidic (∼650 mV per decade) solutions, it is likely that Mn sites in the mixed metal film do not contribute to OER. Overall, there appears to be no catalytic synergy between Co and Mn, as the addition of Mn does not appear to change the electrochemical properties of CoO_*x*_. The advantage of Mn emerges in its role as a structural metal. Stability tests for CoMnO_*x*_ demonstrate continuous oxygen evolution (at 0.1 mA cm^–2^) in pH 2.5 for over 12 h without dissolution (Fig. 5a) and with an OER faradaic efficiency near unity (Fig. S3†). For these stability measurements, constant current density was maintained to ensure a steady rate of catalysis and provide an indicator for film degradation. For stable films, the OER potential remains steady over the course of the experiments. Conversely, as the film dissolves, the potential slowly rises until there is no solid catalyst remaining, whereupon a sharp inflection of potential is observed with the current density approaching that of a blank FTO. Whereas such an inflection is observed for CoO_*x*_ and CoMnO_*x*_ films with <50% Mn composition (which fully degrade within 3 h), MnO_*x*_ and CoMnO_*x*_ films with ≥50% Mn content show no such dissolution behavior as they remain intact over long-term acidic OER (Fig. 5a). Thus, the stability of CoMnO_*x*_ appears to be derived from oxide films possessing a Mn : Co ratio that is equal or greater than unity. It is noteworthy that IrO_*x*_ performs OER at 0.1 mA cm^–2^ in pH 2.5 at a potential that is only 140 mV lower than that of CoMnO_*x*_ films (≥50% Mn).

### CoPbO_*x*_ and CoFePbO_*x*_

**Fig. 13:**
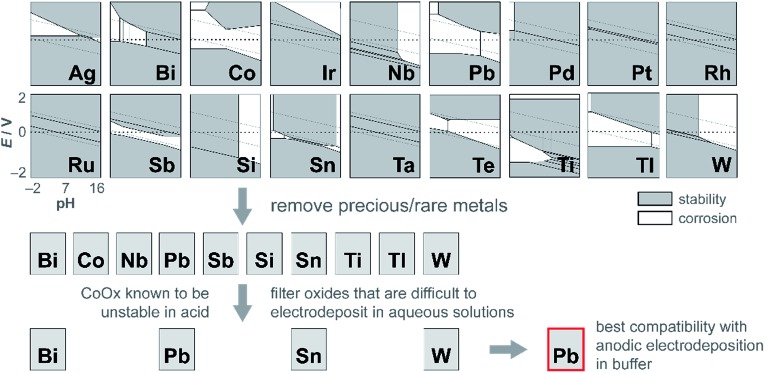
Process for independently optimizing the structural component of mixed metal films to discover a metal oxide that is both stable at high anodic potentials and at acidic pH. Pourbaix diagrams of metals (shown as simplified representations of stability and corrosion, generated from the Materials Project^97^ and experimental data^45^) were analysed for stability in the top left region of the plots (corresponding to high anodic potentials and low pH). The candidates were then filtered by removing precious and rare metals; then further refined by excluding oxides that were incompatible with anodic electrodeposition in buffer. In this manner, Pb was identified as a promising replacement for Mn for stabilizing OER catalysts in acid.

Although CoMnO_*x*_ is active, stable, and composed of earth-abundant elements, Mn-based oxide films have the limitation in that they are oxidized to permanganate ions (MnO_4_^–^),^45,93^ which are soluble and lead to catalyst dissolution at high anodic potentials (see Fig. S8,† Pourbaix diagram). A workaround is to restrict the upper operating potential of these films by operating at low current densities and by increasing the amount of catalyst loading, which may be achieved with the use of porous electrodes.^94–96^ Nonetheless, it is also desirable to support higher operating current densities without resorting to physical surface area augmentation. To this end, we sought to replace Mn with another structural metal that is stable in acid at high anodic potentials. Experimentally^45^ and computationally^97^ derived Pourbaix diagrams of transition and post-transition metals guide in the selection of passivated oxide phases that persist at low pH and high potentials. The initial screen presented 16 candidates (Fig. 13) that were down-selected based on removing elements that are not earth-abundant (*e.g.*, precious metals), experimentally known to be unstable in acid (*e.g.*, Co), and difficult to electrodeposit (*e.g.*, Nb, Ti, and Si). Of the four elements remaining (Bi, Pb, Sn, and W), Pb was the best candidate because it can be anodically electrodeposited easily and has a significant advantage in electrical conductivity owing to its oxygen defects.^98,99^ Acknowledging the toxicity of Pb, this concern is somewhat mitigated inasmuch as a small amount of the element is needed for the mixed metal catalyst; moreover for the purposes of this study, the focus was to assess the viability of an independent catalytic/structural mixed-metal approach. On its own, PbO_*x*_ electrodeposits under similar conditions as CoO_*x*_ but with faster deposition kinetics (as evidenced by the steepness of the Pb^2+^ oxidation wave in Fig. S1e†). PbO_*x*_ exhibits ∼120–130 mV per decade Tafel slopes for OER in neutral and acidic pH.^100^ When compared to CoO_*x*_ under the same conditions, PbO_*x*_ is a relatively poor catalyst and also suffers from a sudden increase to “infinite” slope near 1 mA cm^–2^ for the acidic regime (Fig. 1b), suggesting that additional driving force has no effect on the rate of the reaction. Disregarding its inferior OER properties, PbO_*x*_ is an excellent structural metal for long-term acidic OER stability at higher current densities (of 1 mA cm^–2^, Fig. 5b), with over 12 h of continuous operation without degradation. We note stability measurement conditions are comparable to those made on acidic OER catalysts near pH 0 at ∼3 mA cm^–2^ for at least 2 h. Here a slightly less acidic pH is offset by better buffering conditions (sulfate and phosphate at pH 2 and 2.5, respectively).

Against this backdrop, Pb was employed as the structural element for Co as the catalytic component. Electrodeposition of CoPbO_*x*_ was similar to that for CoMnO_*x*_ with the precursor solution containing equal concentrations of Co^2+^ and Pb^2+^ in MeP_i_ buffer at pH 8. However, the potentials for CoO_*x*_ and PbO_*x*_ deposition are similar (∼1.15 V) unlike that for CoO_*x*_ and MnO_*x*_ deposition; thus, the composition of the mixed metal film could not be as easily controlled through varying the potential. Instead, the initial concentrations of Co^2+^ and Pb^2+^ can be relatively adjusted to change composition. For solutions containing equal concentrations of Co^2+^ and Pb^2+^, electrodeposited CoPbO_*x*_ films contain 18% Co and 82% Pb (as measured by ICP-MS, Table S1†) with homogeneous distribution of the two metals throughout the film (as observed from EDS elemental maps from SEM, Fig. S4c†). The use of CoPbO_*x*_ as a stable electrode has precedence in the electrosynthesis of oxidants, decomposition of organics, as well as electrowinning of metals.^101–103^ Although the application of CoPbO_*x*_ for acidic oxygen evolution has largely been unexplored, its prior history in corrosive environments suggested that it was a promising target.

Like CoMnO_*x*_, the OER performance of CoPbO_*x*_ resembled that of CoO_*x*_ with similar Tafel slope of ∼72 mV per decade in neutral and acidic pH (Fig. 4). While there is no catalytic synergism between Co and Pb, there is no discord either: the presence of Pb does not hinder the OER activity of Co sites. Interestingly, given that the mass loading of the films are comparable, the number of total Co active sites (*i.e.*, 18% Co) in CoPbO_*x*_ should be less than that of CoO_*x*_. However, Tafel plots of CoPbO_*x*_ overlaid with those of CoO_*x*_, which indicates a similar level of activity. This effect could manifest from: first, the difference in morphology of the films where CoO_*x*_ has a smooth surface that may limit the accessibility of active sites while CoPbO_*x*_ is a rougher film comprised of small nanoparticles; and secondly, the high electrical conductivity of PbO_*x*_ may enable a greater number of Co sites to be electrically addressed in CoPbO_*x*_ with low internal resistance. Four-point probe electrical measurements of PbO_2_ exhibit metallic-like bulk resistivity from 10^–3^ to 10^–5^ ohm cm.^104,105^ Conversely, early transition metal oxides generally behave as insulators or semiconductors with high resistivity (in units of ohm cm): Co_3_O_4_ on the order of 10^4^,^106,107^ MnO_2_ from 10^2^ (electrolytic manganese dioxide, EMD) to 10^5^ (birnessite),^108,109^ and Fe_2_O_3_ and FeOOH from 10^4^ to 10^8^.^110,111^ PbO_2_ is orders of magnitude more conductive than Co_3_O_4_ although its exact influence on CoO_*x*_ when co-deposited as CoPbO_*x*_ requires direct conductivity measurements by electrochemical impedance spectroscopy or *via* an interdigitated electrode array. Thus, Pb in the mixed metal film appears to act as a structural metal while Co plays the catalytic role.

The acidic OER stability of CoPbO_*x*_ was evaluated at intermediate (1 mA cm^–2^) current densities (Fig. 5b) where the presence of Pb enhanced the corrosion resistance of the films. For OER at 1 mA cm^–2^, both CoMnO_*x*_ and CoO_*x*_ films fully dissolve within 30 min while CoPbO_*x*_ resists degradation to 7 h. While the addition of Pb does not confer long-term stability against acid corrosion, it does slow the rate of degradation. Thus, the role of Pb as an anodically stable metal in acid was promising and encouraged us to test variants of CoPbO_*x*_.

One such variant involved the use of Co and Fe as the catalytic metal components with Pb remaining as the structural element. The motivation for CoFe stemmed from recent studies demonstrating a synergistic effect that increases the kinetics for OER when Fe is doped into Co or Ni oxides.^51,54,55,70^ We were curious if the low Tafel slope of 30 mV per decade in alkaline pH could translate to neutral and acidic solutions with the aid of Pb for stabilization at lower pH. As a control, Tafel plots of CoFeO_*x*_ (ref. 54) were recorded at neutral and acidic pH; here, Co and Fe did not show any catalytic synergy, as the Tafel plots (with 65–70 mV per decade slope) overlaid with those of CoO_*x*_ (Fig. 4). Stability tests of CoFeO_*x*_ at higher current densities of 1 mA cm^–2^ show improvement in stability with film dissolution at ∼1.5 h instead of at ∼0.5 h for CoO_*x*_ (Fig. 5b). This demonstrates that Fe has a minor structural role, which is consistent with a trend of trading off activity for stability when moving to earlier first-row transition metals. Next, Pb-stabilized CoFeO_*x*_ was anodically electrodeposited in a solution containing equal concentrations of Co^2+^, Fe^2+^, and Pb^2+^. The resulting film exhibited a composition of 15% Co, 2% Fe, and 83% Pb (by ICP-MS, Table S1†) with homogeneous distribution of the three metals (as determined to ∼7.4 Å resolution by EDS elemental maps from SEM and STEM, Fig. 8 and 9). Tafel analysis of CoFePbO_*x*_ in neutral and acidic solutions shows no difference from that of CoO_*x*_ films (slopes of 65–70 mV per decade, Fig. 4). However, unlike CoO_*x*_ and CoFeO_*x*_ catalyst films, CoFePbO_*x*_ exhibits long-term stability in acid, even at higher current densities. While CoPbO_*x*_, CoFeO_*x*_, and CoO_*x*_ dissolve in pH 2.5 buffer when performing OER at of 1 mA cm^–2^, CoFePbO_*x*_ can maintain a steady ∼1.65 V for over 12 h of operation (Fig. 5b) with a faradaic efficiency near unity (Fig. S3†). To further demonstrate the robustness of these films, CoFePbO_*x*_ was continuously operated at 1 mA cm^–2^ for over 50 h in sulfate buffer at pH 2.0 (Fig. 5c) and at only ∼220 mV higher potential than IrO_*x*_ operating in the same conditions (Fig. 5b); little degradation was observed at OER operating voltages of ∼1.8 V.

CoMnO_*x*_ (at 0.1 mA cm^–2^) and CoFePbO_*x*_ (at 1 mA cm^–2^) exhibit chemical stability when performing acidic OER near pH 2.5. There are two important considerations for their operation. First their stability depends on application of an anodic bias above ∼1.3 V during operation to maintain its oxidic state. As known for CoO_*x*_ and as apparent in the Pourbaix diagrams of Co and Pb (Fig. S8†), the Co_3_O_4_ and PbO_2_ states are only maintained at high anodic potentials (above the equilibrium potential for OER) under acidic pH. When voltage is switched off, there is a slow decay of the film back to its original Co^2+^ and Pb^2+^ ions in solution. Second, whereas these films are chemically stable, they not indefinitely stable because they lack the ability to self-heal.^75^ Over long period of operation, mechanical stress on the film from the evolution of O_2_ gas slowly removes catalyst material from the electrode. Because CoFePbO_*x*_ (and also CoMnO_*x*_) cannot be electrodeposited in acidic conditions (their electrosynthesis occurs at near-neutral pH), mechanical losses cannot be repaired during operation. Discovering an OER catalyst that can both electrodeposit and operate efficiently under acidic pH will furnish self-healing and allow mechanical challenges to be overcome, as is the case for native CoP_i_ and NiB_i_ catalysts.

### Nature of stability

The foregoing results demonstrate that structural metals stabilize catalytic sites in mixed metal oxide films. Guidelines emerge for achieving stability while maintaining OER activity. First, mixed metal films should be composed of at least 50% structural metal to maintain an appreciable stability. For example, CoMnO_*x*_ films prepared with ≥50% Mn do not dissolve in pH 2.5 solution (for OER at 0.1 mA cm^–2^) while the film with only 17% Mn degraded quickly, similar to a CoO_*x*_-only film (Fig. 5a). Given the homogeneous distribution of metals in these catalysts, the requirement that at least half of the film is composed of structural metal suggests that stabilization of active sites requires sufficient scaffolding to exist around those sites. Second, Tafel analyses of mixed metal films show no improvement in OER kinetics when compared to the unary catalytic metal oxide films. This is consistent with XPS spectra indicating negligible differences in the electronic features of Co when comparing CoMnO_*x*_ and CoFePbO_*x*_ to CoO_*x*_ (Fig. 10a and 11a), suggesting that the Co catalytic sites are unchanged from the unary system and obey the same catalytic mechanism (*i.e.*, same Tafel slope) in all three systems. As a corollary, the structural metals do not significantly participate in catalysis, as both MnO_*x*_ and PbO_*x*_ have poor OER kinetics and do not affect the Tafel slope of Co catalytic sites in the mixed metal films. However, the structural metal does appear to engage in internal transport of electrons (and possibly protons) inasmuch as even when the number of catalytic sites diminishes in mixed films, its absolute activity (*i.e.*, position of the Tafel plot) remains similar, suggesting that the scaffolding provided by the structural element permeates throughout the film and provides an electronic pathway for addressing active sites.

These results are consistent with the contention that the structural metal provides an oxide that embeds the catalytic element and prevents it from dissolution. After OER, the metal–oxygen framework is disrupted with the elimination of O_2_, and the formal oxidation state of the catalytic metal is reduced, resulting in a weakening of the metal–oxygen framework owing to population of metal–oxygen antibonding orbitals and an attendant decrease in the ligand field strength afforded by moving from oxo to hydroxyl/water.^60–62,112,113^ The presence of structural metals helps fortify the oxygen framework about the catalytic active site thus minimizing its degradation and leading to enhanced stability of the mixed-metal oxide. This fortification of the oxygen framework stems from the lack of catalysis at structural sites and thus preservation of the structural metal at a high formal charge, as indicated by XPS spectra on CoMnO_*x*_ (Fig. 10) and CoPbO_*x*_/CoFePbO_*x*_ (Fig. 11). For the former, XPS spectra indicates a slight broadening of the Mn 2p peaks to incorporate features of higher binding energies relative to that of MnO_*x*_ (Fig. 10b). Given that as-deposited MnO_*x*_ is birnessite-like (δ-MnO_2_), which has an average Mn oxidation state of +3.8, the shifting of Mn 2p peaks to higher binding energies suggests an increase in Mn oxidation state nearer to +4.0, which may reflect a pyrolusite-like phase (β-MnO_2_).^25,82,83^ Similarly, for CoPbO_*x*_ and CoFePbO_*x*_, the shift of the Pb 4f peaks to higher binding energies when compared to PbO_*x*_ implies the stabilization of the PbO_2_ phase.^59^ It is important to maintain this phase because the reduction of PbO_2_ to Pb^2+^ can occur readily at low pH (as governed by its Pourbaix diagram).^45^ Thus the increase in binding energy, especially from the doping of Fe, corresponds to a slightly higher average formal oxidation state of Pb, which provides additional anodic protection of PbO_*x*_ against corrosion. We believe that while catalytic sites undergo redox state changes during OER that can momentarily weaken the metal–oxo bond, the oxidation state and bonding environment of the structural metal remains consistent and provides the electron and proton conductivity to facilitate re-oxidization of catalytic species before dissolution.

## Conclusion

The current status of acidic OER catalyst properties can be summarized by picking any two properties from highly active, stable, and comprised of earth-abundant materials. Commercial catalysts use Ir or Ru oxides, which are expensive and not scalable, while the best earth-abundant catalysts (such as Co and Ni oxides) dissolve in acid. We demonstrate here that catalysts may be iteratively designed to fulfill the three properties for acidic OER. Unary metal oxides trade activity for acid stability. This trade-off may be circumvented by decoupling catalytic and structural sites as separate elements in mixed metal oxide films. Combining Co as the catalytic metal with Mn as the structural element engenders fast OER kinetics for CoMnO_*x*_ in acid (∼82 mV per decade Tafel slope in pH 2.5) with sustained activity of 0.1 mA cm^–2^ for over 12 h; this behavior contrasts CoO_*x*_, which dissolves in minutes under the same conditions. To operate at higher current densities, Pb serves as the structural element in CoPbO_*x*_ and CoFePbO_*x*_ films, which maintain the OER activity of Co while displaying resistance to corrosion in pH 2.5 solution. Whereas CoO_*x*_ dissolves within 30 min, CoPbO_*x*_ is stable for 7 h and CoFePbO_*x*_ does not degrade after 12 h. Furthermore, CoFePbO_*x*_ can sustain OER current densities of 1 mA cm^–2^ for 50 h at pH 2.0. This result is noteworthy in the context that the activity of CoFePbO_*x*_ comprising earth-abundant elements occurs at ∼220 mV higher overpotential than that of IrO_*x*_ operating at 1 mA cm^–2^ in pH 2.5 solution. In this regard, the principle of using different metals to fulfil independent functional components within extended lattices may be useful for designing catalysts that need to fulfil several different criteria simultaneously.

## Experimental

### General electrochemical details

Electrochemical experiments were conducted on a CH Instruments 760D potentiostat. A three-electrode configuration (working, reference, and auxiliary) in glass H-cells was employed where a porous glass frit separated the working and auxiliary compartments. Glassware was pre-cleaned by soaking in aqua regia followed by type I reagent water (EMD Millipore, 18 MΩ cm resistivity). The working electrode was fluorine-doped, tin oxide-coated glass (FTO) with 7 Ω sq^–1^. surface resistivity (TEC-7, precut 1 cm × 2.5 cm slides from Hartford Glass). Prior to use, FTO slides were cleaned by sonication in acetone and then rinsing with type I water; a 1 cm^2^ geometric electrode area was created by masking the FTO with Scotch tape, which was removed immediately after film electrodeposition. A Ag/AgCl reference electrode (BASi, filled with saturated KCl) was positioned close to the FTO in the working compartment, and a Pt mesh (99.9%, Alfa Aesar) electrode in the auxiliary side of the H-cell was used to complete the circuit. All experiments were conducted at ambient temperature (∼23 °C). Uncompensated resistance was first measured on a blank FTO electrode in the same cell setup and then corrected through automatic positive feedback during cyclic voltammograms or by subtracting the ohmic potential drop from applied potentials in Tafel and stability measurements after data collection. Potentials were converted to the NHE scale by the following relation: *E*_NHE_ = *E*_Ag/AgCl_ + 0.197 V, and overpotentials (*η*) for the oxygen evolution reaction from water were calculated by: *η* = *E*_OER_ – (1.23 V – 0.059 V × pH). Positive potentials are oxidizing while negative potentials are reducing.

### Electrodeposition of catalyst films

Catalysts were prepared by anodic electrodeposition. Generally, a constant anodic potential is applied to 1 cm^2^ FTO for a fixed amount of time in a 50 mM methylphosphonate (MeP_i_) solution buffered at pH 8.0 containing a total of 0.5 mM metal ion(s). MeP_i_ was prepared from methylphosphonic acid (98%, Alfa Aesar) that was purified by recrystallizing twice from boiling acetonitrile (HPLC grade, Aldrich). Although MeP_i_ is a preferred buffer since it stabilizes metal ions in solution (yet does not bind too strongly to precipitate the complex) and buffers well in slightly alkaline pH (a regime that accommodates the anodic electrodeposition of a wide range of candidate metal ions), alternative buffers such as acetate at neutral pH also suffice. pH of the buffer solutions was adjusted with KOH (<0.001% Ni, Fe, and other heavy metals; from EMD Millipore). After deposition, films were briefly immersed in type I water to remove any lingering trace metal ions and subsequent electrochemical characterization was performed immediately to prevent films from drying.

Electrodeposition conditions for unary metal oxide films employed previous literature procedures in the presence of 50 mM MeP_i_ at pH 8.0: MnO_*x*_ from 0.5 mM Mn^2+^ (MnCl_2_·4H_2_O, 99.995% trace metal basis, Strem) at 0.54 V;^24^ cobalt oxide (CoO_*x*_) from 0.5 mM Co^2+^ (using Co(NO_3_)_2_·6H_2_O, 99.999% trace metal basis, Strem) at 1.05 V;^46^ nickel oxide (NiO_*x*_) from 0.5 mM Ni^2+^ (using Ni(NO_3_)_2_·6H_2_O, 99.9988% trace metal basis, Strem) at 1.25 V;^18^ and lead oxide (PbO_*x*_) from 0.5 mM Pb^2+^ (using Pb(NO_3_)_2_, 99.999% trace metal basis, Strem) at 1.35 V.^47^ Iron oxide (FeO_*x*_) was electrodeposited at 1.20 V in a solution of 0.5 mM Fe^2+^ (using (NH_4_)_2_Fe(SO_4_)_2_·6H_2_O, 99.997% trace metal basis, Aldrich) with 1.0 M KNO_3_ (99.0–100.5%, Macron) heated to 75 °C.^48^ For comparison, iridium oxide (IrO_*x*_) was prepared following literature procedures at 0.85 V in 2 mM Ir^3+^ (from K_3_IrCl_6_, Aldrich) with 15 mM oxalate (from oxalic acid, 98.9–101.0%, Aldrich) in 100 mM carbonate buffer (from K_2_CO_3_, >99.0%, Aldrich) at pH 10.5.^49^ Cobalt-iron oxide (CoFeO_*x*_) films were cathodically electrodeposited from *ca.* 1 mM Co^2+^ and Fe^2+^ at –0.3 V, similar to literature procedures.^54^ Mixed metal oxide films were also anodically co-deposited in MeP_i_ buffer at pH 8.0: CoMnO_*x*_ at 0.65, 0.90, and 1.15 V in *ca.* 0.25 mM of Co^2+^ and Mn^2+^; CoPbO_*x*_ at 1.15 V in *ca.* 0.25 mM Co^2+^ and Pb^2+^; and CoFePbO_*x*_ at 1.15 V in *ca.* 0.125 mM Co^2+^ and Pb^2+^ with 0.25 mM Fe^2+^. All deposition protocols aimed to achieve roughly similar mass loading of films.

### Tafel data collection

The oxygen evolution activity of catalyst films was evaluated by measuring the steady-state current density (*j*) as a function of applied potential (*E*) in buffered solutions of 100 mM phosphate (P_i_, prepared from phosphoric acid, 99.99% trace metal basis, Aldrich) and 1.0 M KNO_3_ at neutral (pH 7.0) and acidic (pH 2.5) conditions. Steady-state conditions were achieved by holding films at each discrete potential for at least 30 s to allow the current to converge, and measurements were initiated at the highest potential (the first point in the series) for at least 100 s to further minimize any pseudocapacitance. Solutions were stirred at ∼600 rpm with a Teflon stir bar (sufficient to remove mass transport limitations) and applied potentials were post-corrected for uncompensated resistance by subtracting *iR* (measured on a blank FTO in same solution conditions) from each point where typical values of uncompensated resistance are ∼17 Ω at pH 7 and 2.5. Further precautions were exercised by targeting Tafel data collection at current densities between 1 μA cm^–2^ and 1 mA cm^–2^ where these lower currents minimize the impact of any ohmic drop. The current–potential data were plotted as log *j vs. E* (or *η*, the overpotential) to construct Tafel plots, where the position and slope (within 5 mV per decade) of independently prepared films under the same conditions were reproducible.

### Acid stability during oxygen evolution

Catalyst degradation during oxygen evolution in acidic pH was assessed by long-term chronoamperometry. Similar conditions were employed as for Tafel measurements but with higher concentration of buffer to decrease local pH gradients in the H-cell during prolonged electrolysis: 0.5 M P_i_ at pH 2.5 and stirred at ∼600 rpm. Chronoamperometry on catalyst films was performed at 0.1 mA cm^–2^ and 1.0 mA cm^–2^, and the potential was recorded over time until films were completely dissolved or until 12 h had passed. Dissolution was indicated visually (*i.e.*, the FTO becomes transparent) and also by the inflection in potential that sharply increases to resemble the catalytic properties of blank FTO or catalysis by metal ions in solution. As a demonstration, CoFePbO_*x*_ was also evaluated over longer time and at slightly lower pH in 0.5 M sulfate (from sulfuric acid, 99.99% trace metal basis, Aldrich) at pH 2.0 for over 50 h of continuous operation. Independently prepared samples following the same protocol were reproducible within 20 min of stability time.

### Faradaic efficiency of oxygen evolution

The faradaic efficiency of oxygen evolution on CoMnO_*x*_ and CoFePbO_*x*_ was determined in 0.5 M P_i_ at pH 2.5 using a gas chromatograph. The films were mounted in a custom-built two-compartment electrochemical cell where a cation-exchange membrane (Nafion 117, Sigma Aldrich) was used to separate the two chambers. A Ag/AgCl-based leak-free reference electrode (LF-1, Warner Instruments) was used as the reference electrode and a Pt wire was the counter electrode. A Viton O-ring was applied to define the area of working electrode and OER was sustained at constant current densities of 0.1 and 1 mA cm^–2^ for CoMnO_*x*_ and CoFePbO_*x*_, respectively. While stirring, a constant flow of Ar gas (20 sccm) was bubbled through the chamber of working/reference electrodes. The gas outlet was connected to a gas chromatograph equipped with a thermal conductivity detector (multiple gas analyzer #3, SRI Instruments). The amount of O_2_ in the out-fluxing Ar gas was quantified, based on the calibration with known O_2_ concentrations. Initial control experiments were performed to ensure that the O_2_ in the air has no contribution to the measured O_2_ signals. The detected O_2_ concentrations were compared to the theoretical yield of O_2_ (calculated by dividing the charge passed by 4*F*) to obtain the faradaic efficiency.

### Inductively coupled plasma mass spectrometry (ICP-MS)

Trace elemental analysis on catalyst films was performed with a quadrupole ICP-MS (Thermo Electron, X-Series ICP-MS with CCT). Because these films are resistant to passive dissolution in acid, they were digested by voltage cycling (between 1.3 and –0.4 V) in 12 mL of 2% v/v nitric acid (TraceSELECT, Fluka). Co, Mn, Pb, and Fe calibration standards were prepared from corresponding ICP standard solutions (TraceCERT, Fluka), which enabled the construction of a calibration curve to convert the detected counts for ^59^Co, ^55^Mn, ^208^Pb, and ^57^Fe to concentrations (in ppm).

### Electron microscopy

The compositional morphology of catalyst films was observed by field emission scanning electron microscopy (SEM, Zeiss Supra 55VP) operated at a beam voltage of 15.0 kV, working distance of ∼8.5 mm, a 30 μm aperture, and an InLens detector. Elemental quantification was determined at a beam voltage of 14 kV with an energy dispersive X-ray spectrometer (EDS from EDAX Inc.) using EDAX ZAF correction factors. Homogeneity on the 20 nm per px scale of mixed metal films were evaluated by EDS elemental maps using characteristic X-rays at the K-edge for Co, Mn, and Fe as well as the M-edge for Pb.

CoMnO_*x*_ and CoFePbO_*x*_ samples were subjected to higher resolution examination of crystallinity and homogeneity by using spherical aberration corrected high angle annular dark field scanning transmission electron microscopes (JEOL ARM200F HAADF STEM and Hitachi HD-2300 STEM). Samples were directly electrodeposited on 400-mesh pure carbon film supported on Au TEM grids (Electron Microscopy Sciences) by partially immersing the grid in the deposition solution as the working electrode. Samples were then dipped in type I water to remove any residual electrolyte and dried at room temperature. Images were taken at a beam accelerating voltage of 200 kV in the scanning transmission electron mode. Brightness and contrast of the images were processed using Gatan DigitalMicrograph software. Homogeneity on the 7.4 Å per px scale of mixed metal films were evaluated by EDS elemental maps using signals corresponding to K line energy values for cobalt, manganese, and iron; and L line energy values for lead.

### X-ray photoelectron spectroscopy (XPS)

Comparison of oxidation state and chemical environment between unary and mixed metal catalyst films employed XPS (using a Thermo Scientific K-alpha XPS system). Catalyst films were electrodeposited on FTO as described above for: CoFePbO_*x*_, CoPbO_*x*_, CoMnO_*x*_, CoO_*x*_, PbO_*x*_, and MnO_*x*_. All samples were illuminated using a monochromated Al Kα X-ray source (1486.6 eV energy and 0.85 eV line width)^114^ with a 400 μm spot size. Surface charging was compensated by a low-energy (0–14 eV) electron flood gun. The system was pre-calibrated with Au, Ag, and Cu standards built into the sample stage using an automated routine. High-resolution spectra for Co 2p, Mn 2p, Pb 4f, and Fe 2p were measured with a step size of 0.1 eV. All spectra were then calibrated to the C 1s peak at 284.8 eV.^57^

### Powder X-ray diffraction (XRD)

The crystallinity of CoMnO_*x*_ and CoFePbO_*x*_ samples were tested by XRD. To maximize the possibility of observing diffraction, powder samples of the catalysts were prepared by large-scale electrodeposition (using the same protocols described earlier) on multiple 20 × 8 cm^2^ FTO plates. The thin films were briefly immersed in type I water to remove any residual solution, dried, carefully stripped from the FTO, and ground into a fine powder. The powders were loaded in a small cavity in a Si zero-diffraction plate (MTI Corporation) and inserted into a Bruker D2 Phaser powder diffractometer equipped with a Cu Kα X-ray source (generated at 20 kV and 20 mA; passed through a 1 mm slit) and LynxEye detector. A knife-edge attachment was used to reduce scattered signal, and the stage was rotated by 3° min^–1^. Samples were examined in Bragg–Brentano mode from 2*θ* = 10 to 75° in 0.02° increments with 0.5 s per point scan rate.
