# Supplementary material for: Design of template-stabilized active and earth-abundant oxygen evolution catalysts in acid
Source: Chem Sci. 2017 May 5;8(7):4779–94. doi: 10.1039/c7sc01239j (PMC5637126; doi:10.1039/c7sc01239j)
Supplement: Supplementary file 1 [file SC-008-C7SC01239J-s001.pdf]

Electronic Supplementary Material (ESI) for Chemical Science.
This journal is © The Royal Society of Chemistry 2017

Supporting Information

Design of template-stabilized active and earth-abundant oxygen evolution catalysts in acid

Michael Huynh, Tuncay Ozel, Chong Liu, Eric C. Lau, Daniel G. Nocera*

[a]*Department of Chemistry and Chemical Biology, Harvard University, Cambridge, MA 02138, United States.*

dnocera@fas.harvard.edu

Index		*Page*
**Figure S1.**	CVs of Co^{2+}, Ni^{2+}, Fe^{2+}, Mn^{2+}, Pb^{2+}, and Ir^{3+}	S3
**Figure S2.**	OER stability of unary metal oxides in P_i at pH 2.5	S4
**Figure S3.**	Faradaic efficiency of OER on CoMnOx and CoFePbOx by GC	S5
**Figure S4.**	EDS elemental maps of CoMnOx (0.65 and 1.15 V) and CoPbOx	S6
**Figure S5.**	STEM images of CoMnOx and CoFePbOx	S7
**Figure S6.**	PXRD patterns of CoMnOx and CoFePbOx	S8
**Figure S7.**	High-resolution XPS of Fe 2p comparing CoFePbOx and Fe_2O_3	S9
**Figure S8.**	Pourbaix diagrams of Co, Mn, Pb, and Fe oxides	S10
**Table S1.**	Elemental analysis of CoMnOx, CoPbOx, and CoFePbOx films	S11

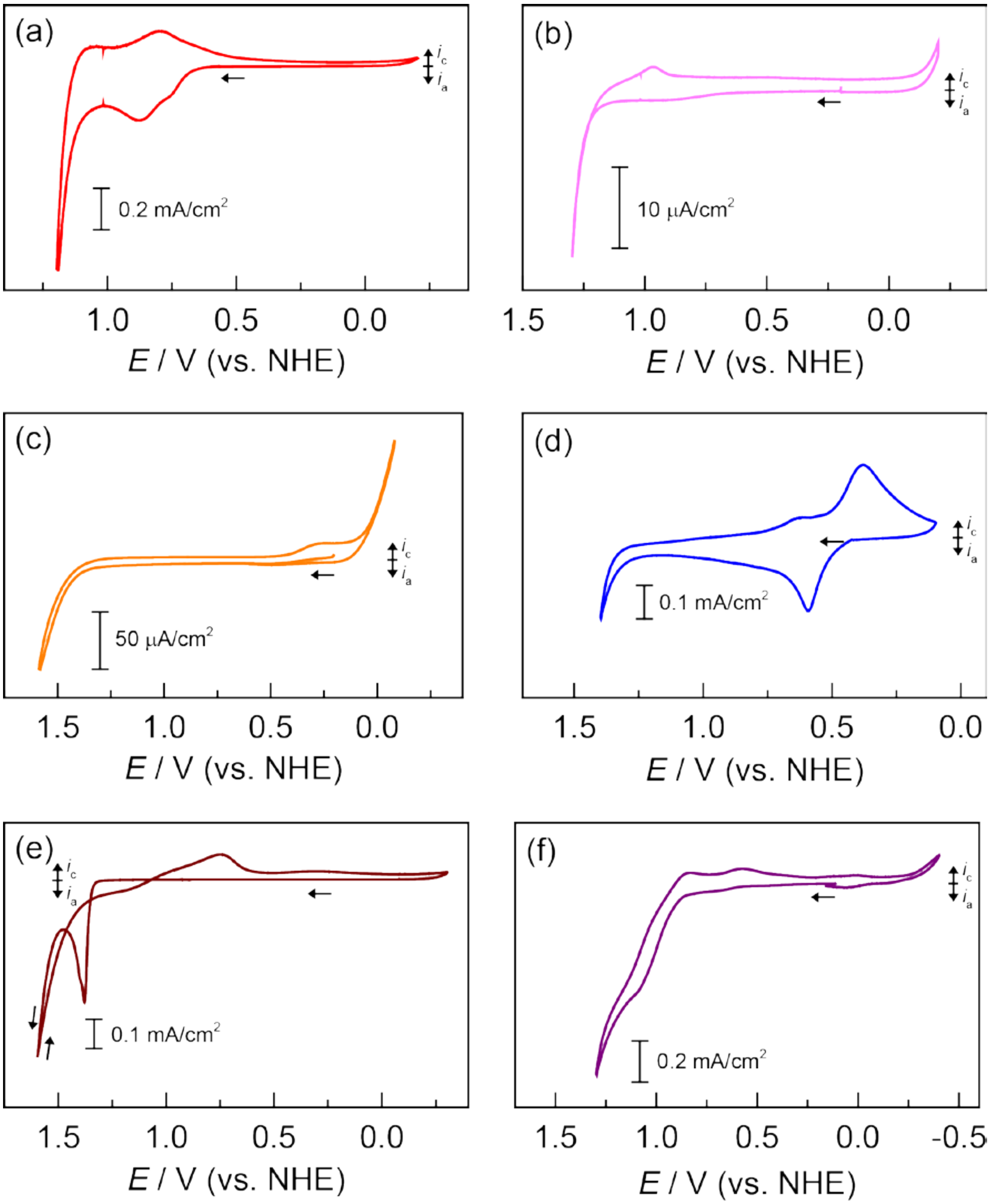

Figure S1. Cyclic voltammograms of a 1 cm^2 FTO electrode at 50 mV/s in 0.5 mM of: (a) Co^{2+} (red ▬), (b) Ni^{2+} (light magenta ▬), (c) Fe^{2+} (orange ▬), (d) Mn^{2+} (blue ▬), (e) Pb^{2+} (brown ▬), and (f) Ir^{3+} (purple ▬). All metal ions were buffered with 50 mM MeP_i at pH 8.0 except for Fe^{2+} (which was deposited at 75 °C in 1.0 M KNO_3) and Ir^{3+} (which was deposited in an oxalate/carbonate buffer at pH 10.5).

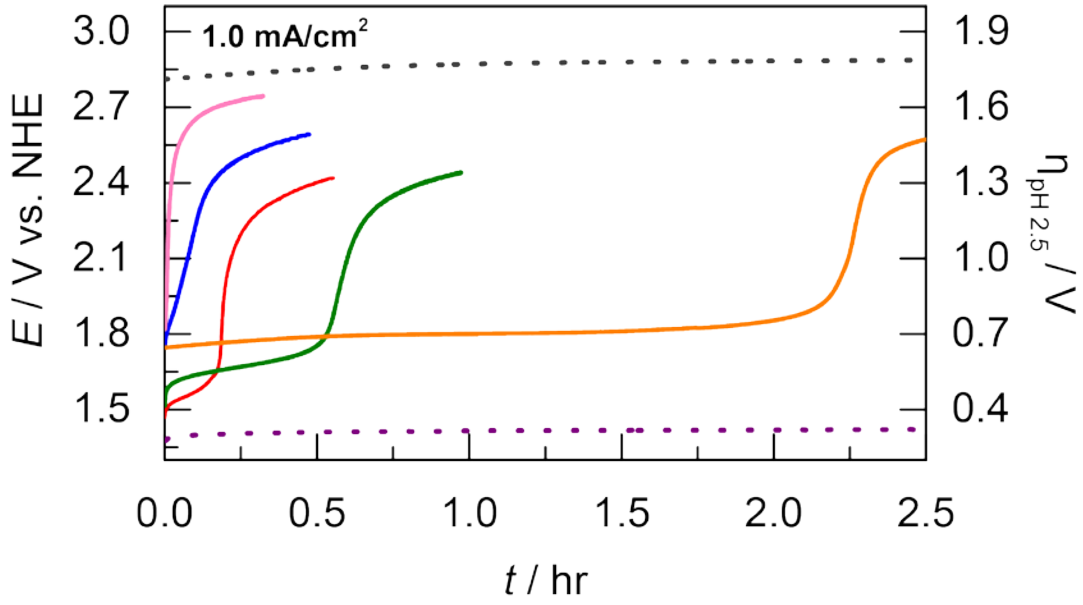

Figure S2. Electrochemical stability for acidic OER measured by sustained chronoamperometry at 1.0 mA/cm^2 in 0.10 M P_i and 1.0 M KNO_3 solution at pH 2.5 for: MnCoOx deposited at 0.90 V (dark green ▬), CoOx (red ▬), MnOx (blue ▬), FeOx (orange ▬), NiOx (light magenta ▬), IrOx (purple •••), and FTO (grey •••). The inflection of potential in the plots indicates film dissolution.

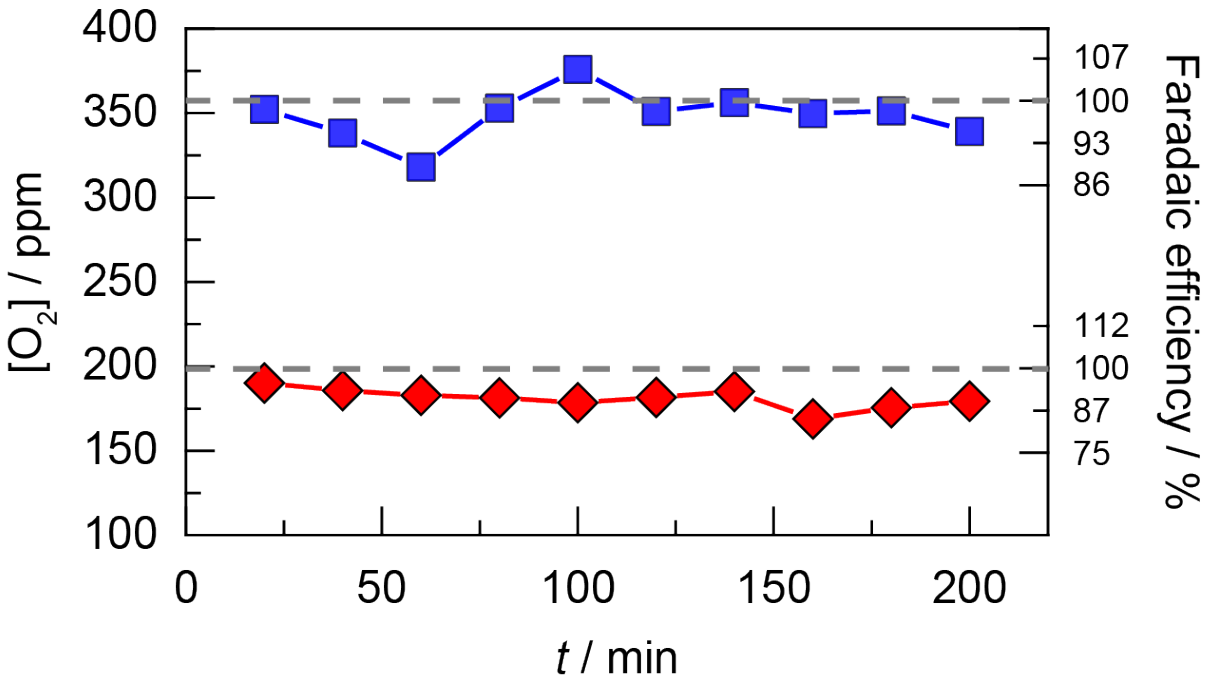

Figure S3. Measured oxygen concentration and corresponding faradaic efficiency of OER in 0.5 M P_i solution at pH 2.5 on CoMnOx operated at 0.1 mA/cm^2 (red, ◆) and CoFePbOx operated at 1.0 mA/cm^2 (blue, ■). O_2 was detected by gas chromatography in 20 min snapshots and theoretical O_2 concentrations (grey, ▬ ▬) are calculated from the charge passed during chronoamperometry assuming 100% Faradaic efficiency. The average efficiency for CoMnOx and CoFePbOx is 91 and 97%, respectively.

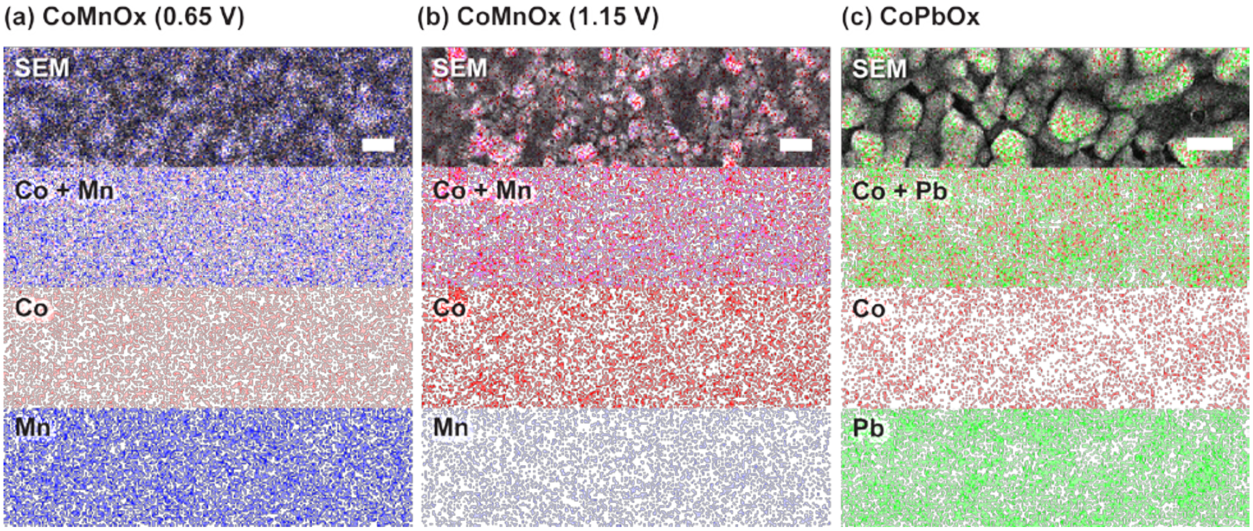

Figure S4. EDS elemental maps recorded through SEM of: (a) CoMnOx (deposited at 0.65 V), (b) CoMnOx (deposited at 1.15 V), and (c) CoPbOx. Individual elemental channels for Co (red), Mn (blue), and Pb (green) were combined and overlaid on the respective SEM image. All samples were prepared on FTO substrate, and scale bars are 200 nm for CoMnOx and 100 nm for CoPbOx.

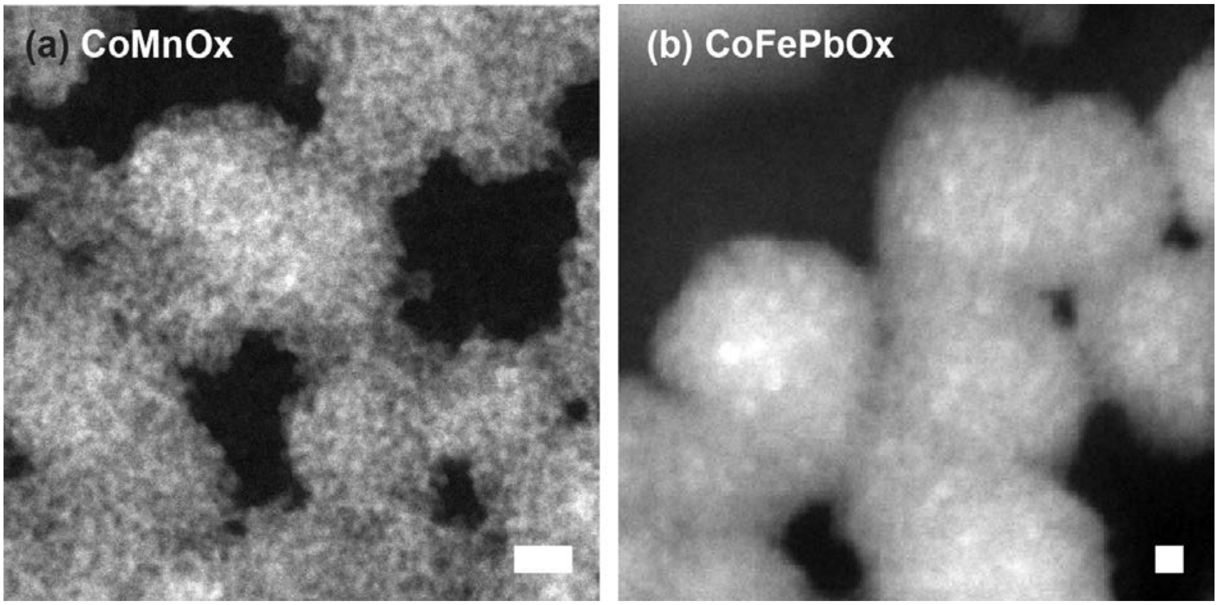

Figure S5. STEM image of (a) CoMnOx and (b) CoFePbOx electrodeposited directly on a carbon mesh TEM grid. Scale bar is 10 nm.

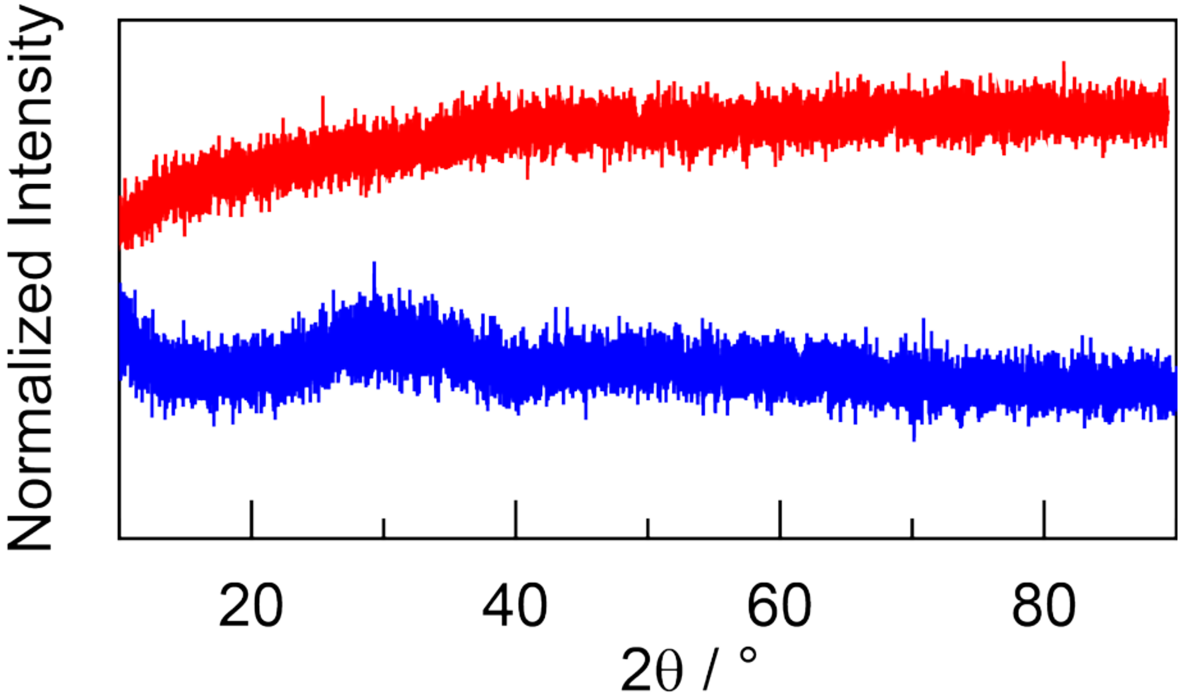

Figure S6. X-ray diffraction patterns of CoMnOx (red, ━) and CoFePbOx (blue, ━) powders, originally prepared as thin films on FTO.

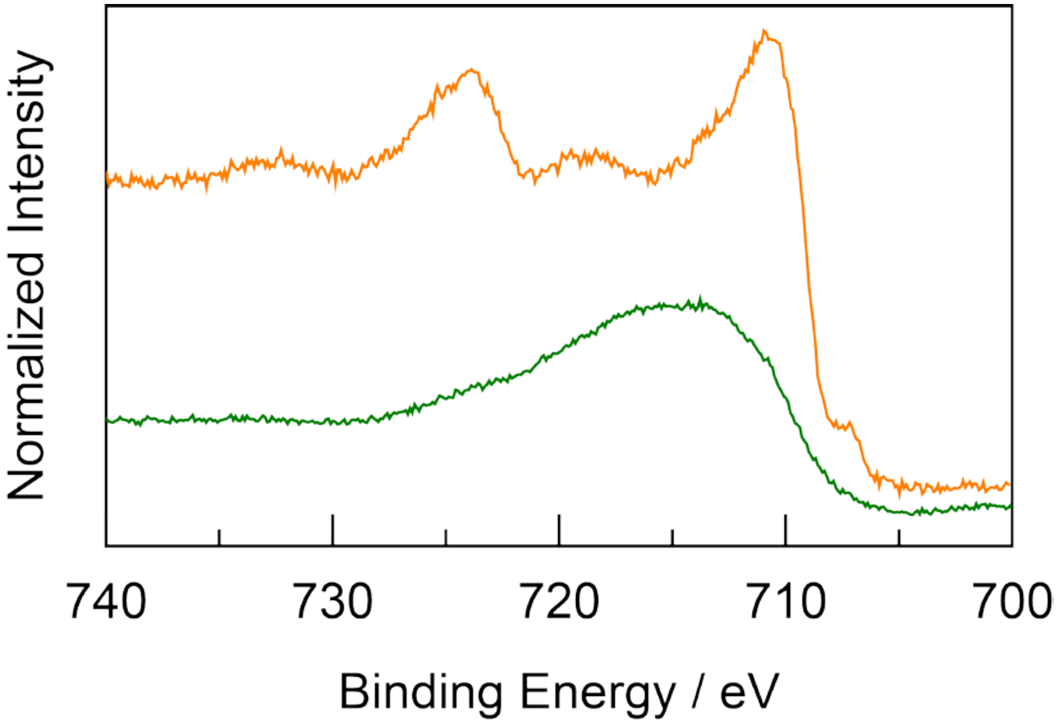

Figure S7. High-resolution XPS spectra in the Fe 2p region for CoFePbOx (dark green —) compared to a Fe_2O_3 control (orange —). The peak for CoFePbOx is assigned to Sn $3p_{3/2}$ from the underlying FTO substrate.

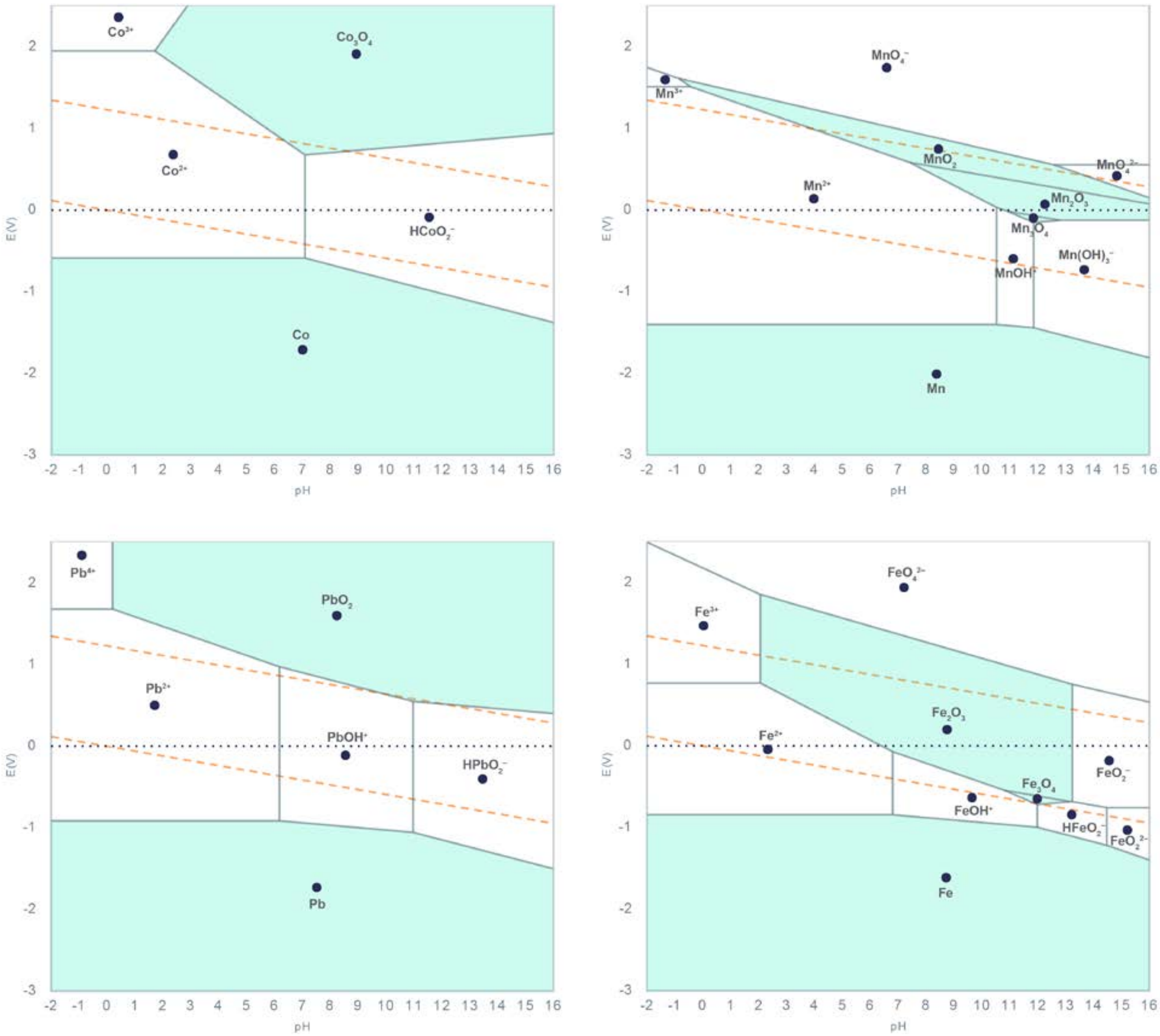

Figure S8. Pourbaix diagrams of Co, Mn, Pb, and Fe oxides generated from the Materials Project.[1]

[1] Jain, A.; Ong, S. P.; Hautier, G.; Chen, W.; Richards, W. D.; Dacek, S.; Cholia, S.; Gunter, D.; Skinner, D.; Ceder, G.; Persson, K. A. *APL Mater.* **2013**, *1*, 011002, materialsproject.org.

Table S1. Elemental analysis of CoMnOx, CoPbOx, and CoFePbOx films.

Sample	Technique[b]	Co %	Mn %	Pb %	Fe %
CoMnOx (0.65 V)[a]	EDS	39	61	0	0
CoMnOx (0.90 V)	EDS	50	50	0	0
	ICP-MS	51	49	0	0
CoMnOx (1.15 V)	EDS	83	17	0	0
CoPbOx	ICP-MS	18	0	82	0
CoFePbOx	ICP-MS	15	0	83	2

[a] Deposition potential of the mixed metal film. [b] EDS denotes energy dispersive X-ray spectrometer employed during scanning electron microscopy. ICP-MS denotes inductively coupled plasma mass spectrometry.
